# Mutate and observe: utilizing deep neural networks to investigate the impact of mutations on translation initiation

**DOI:** 10.1093/bioinformatics/btad338

**Published:** 2023-05-24

**Authors:** Utku Ozbulak, Hyun Jung Lee, Jasper Zuallaert, Wesley De Neve, Stephen Depuydt, Joris Vankerschaver

**Affiliations:** Department of Applied Mathematics, Computer Science and Statistics, Ghent University, Ghent, Belgium; Center for Biosystems and Biotech Data Science, Ghent University Global Campus, Incheon, South Korea; Center for Biosystems and Biotech Data Science, Ghent University Global Campus, Incheon, South Korea; Center for Medical Biotechnology, VIB, Ghent, Belgium; Department of Biomolecular Medicine, Ghent University, Ghent, Belgium; Center for Biosystems and Biotech Data Science, Ghent University Global Campus, Incheon, South Korea; Department of Electronics and Information Systems, Ghent University, Ghent, Belgium; Lab of Plant Growth Analysis, Ghent University Global Campus, Incheon, South Korea; Department of Plant Biotechnology and Bioinformatics, Ghent University, Ghent, Belgium; Erasmus Brussels University of Applied Sciences and Arts, Brussels, Belgium; Department of Applied Mathematics, Computer Science and Statistics, Ghent University, Ghent, Belgium; Center for Biosystems and Biotech Data Science, Ghent University Global Campus, Incheon, South Korea

## Abstract

**Motivation:**

The primary regulatory step for protein synthesis is translation initiation, which makes it one of the fundamental steps in the central dogma of molecular biology. In recent years, a number of approaches relying on deep neural networks (DNNs) have demonstrated superb results for predicting translation initiation sites. These state-of-the art results indicate that DNNs are indeed capable of learning complex features that are relevant to the process of translation. Unfortunately, most of those research efforts that employ DNNs only provide shallow insights into the decision-making processes of the trained models and lack highly sought-after novel biologically relevant observations.

**Results:**

By improving upon the state-of-the-art DNNs and large-scale human genomic datasets in the area of translation initiation, we propose an innovative computational methodology to get neural networks to explain what was learned from data. Our methodology, which relies on *in silico* point mutations, reveals that DNNs trained for translation initiation site detection correctly identify well-established biological signals relevant to translation, including (i) the importance of the Kozak sequence, (ii) the damaging consequences of ATG mutations in the 5′-untranslated region, (iii) the detrimental effect of premature stop codons in the coding region, and (iv) the relative insignificance of cytosine mutations for translation. Furthermore, we delve deeper into the Beta-globin gene and investigate various mutations that lead to the Beta thalassemia disorder. Finally, we conclude our work by laying out a number of novel observations regarding mutations and translation initiation.

**Availability and implementation:**

For data, models, and code, visit github.com/utkuozbulak/mutate-and-observe.

## 1 Introduction

Discovering the secrets of biological systems has been one of the primary goals of humanity in the past couple of centuries. Thanks to the continuous development of sequencing techniques, among other technological advances, we have accumulated large amounts of genomic data that facilitate a better understanding of a variety of biological tasks. One such task is translation initiation, which starts with the assembly of the complete 80S ribosome on the mRNA at the translation initiation codon (canonically, an AUG triplet) (Jackson *et al.* 2010). Since translation initiation is the primary regulatory step for protein synthesis, understanding the mechanisms that influence this step is of utmost importance to understanding translational control in cells. This in turn might impact the discovery of the genetic basis of numerous inherited disorders, with the latter often emerging from dysfunctional or inappropriate translation (Kong and Lasko 2012).

For eukaryotes, initial attempts at translation initiation site (TIS) detection were based on determining consensus scores, and in particular, on the recognition of the Kozak consensus sequence (Kozak 1987, 1989). Until recently, these methods were dominant for the purpose of TIS detection, mainly thanks to their simplicity and effectiveness (Ingolia *et al.* 2009). However, recent research efforts suggest that TIS recognition by the small ribosomal subunit (SSU) is more complicated than initially thought, involving at least nine eukaryotic initiation factors (eIF), as well as other translation factors (Lee *et al.* 2012, Gao *et al.* 2015). Naturally, these findings called for more complex approaches towards TIS recognition within lengthy DNA sequences, resulting in the development of statistical and computational methods for TIS identification (Saeys *et al.* 2007), and with these methods also taking advantage of the rapid increase in the amount of sequenced genomic data.

Recently, advances made in the field of machine learning enabled the usage of deep neural networks (DNNs) for addressing a variety of sequence-based tasks. In fact, the usage of DNNs has become so widespread in the field of computational biology that most of the state-of-the-art methods rely on a variation of this technology (employing convolutional, recurrent, or transformer architectures, to name a few) (Jaganathan *et al.* 2019, Pavlov and Poptsova 2020, Strauch *et al.* 2022). Even though DNN-based methods for TIS detection were adopted in the past with relatively poor success rates (Pedersen and Nielsen 1997, Hatzigeorgiou 2002), such approaches were recently shown to achieve state-of-the-art results (Magana-Mora *et al.* 2013, Zhang *et al.* 2017, Zuallaert *et al.* 2018, Wei *et al.* 2021a). Despite their applicability in genomics, understanding the rationale behind the predictions made by DNNs has been a challenging task. This can be attributed primarily to the black-box nature of DNNs, which hides their complex decision-making abilities. Unfortunately, the lack of insight into the predictive abilities of DNNs induces distrust in the features learned by these models, since these features cannot be verified easily, and eventually, they make DNNs unreliable when the aim is to discover highly sought-after novel biological knowledge. As a result, research efforts that showcase state-of-the-art results in DNN-based TIS identification are either devoid of any discussion on the learned biological features or only contain shallow discussions on a number of well-known features, such as the importance of the Kozak consensus sequence (as detailed above).

To demystify biological signals relevant to translation, we employ point mutations in conjunction with DNNs, where point mutations refer to changes to a single nucleotide. In the past, such mutations were successfully employed by numerous studies to understand the process of translation and to identify genetic disorders (Baliga 2001, De Falco *et al.* 2002, Dvir *et al.* 2013, Inui *et al.* 2014, Zhu *et al.* 2020), suggesting that the impact of mutations on translation may be dramatic, ranging from reduced gene expression levels to the synthesis of a completely different protein (Lazarus *et al.* 2014, Zhang and Lupski 2015).

Mutations to the start codon, in particular, are detrimental to gene expression in the majority of cases. However, in some cases, the presence of a nearby start codon (among other things) can alleviate the effect of such mutations, allowing the variation to subsist in the population (Abad-Navarro *et al.* 2018, Castell-Diaz *et al.* 2022).

Interestingly, the impact of certain point mutations upon biological signaling closely resembles a recently discovered phenomenon in machine learning, namely the existence of so-called adversarial examples (Goodfellow *et al.* 2015). Adversarial examples are maliciously created data points, often through the adoption of small modifications in the input, and where these small modifications cause large changes in the predictions. Such data points are particularly threatening for DNNs in the field of biomedical analysis due to concerns regarding prediction integrity and security (Ozbulak *et al.* 2019). Although such data points are first and foremost a threat in terms of security, a number of studies revealed that it is possible to leverage them for the purpose of DNN interpretability (Pezeshkpour *et al.* 2019, Tao *et al.* 2018), for example, to understand protein structures, which makes them relevant to our study (Carbone *et al.* 2022).

Although similar phenomena in both the field of genomics and the field of machine learning emerged in two different forms, their impact on the integrity of certain processes inspired us to investigate the adversarial nature of mutations in the context of translation. Taking inspiration from the above studies, we propose a computational methodology that combines point mutations with state-of-the-art DNNs that are able to perform TIS detection in human genomes, with the goal of identifying biologically relevant features and discovering novel insights that can be used to guide future research efforts. Different from past research efforts that utilized DNNs for the task of TIS detection, which mostly discuss the relevance of the Kozak consensus sequence to translation, we observe that our model learns three additional well-documented biological signals: (i) the presence of an ATG codon in the 5′-untranslated region (UTR), which may prevent the detection of the authentic start of translation initiation, (ii) the detrimental effects of mutations that lead to the creation of premature stop codons in the coding region, and (iii) the relative insignificance of C mutations to the process of translation. After confirming the biological plausibility of our model, we extract novel biological insights by investigating silent mutations in the 5′-UTR, since mutations in this region attracted a significant amount of research attention in recent years (Tan 2020). Specifically, we identify different types of mutations in the 5′-UTR that may be damaging to translation in humans and that merit further (experimental) validation.

## 2 Materials and methods

### 2.1 Data

To facilitate a comparison with previous research efforts, we make use of the CCDS and Chromosome-21 TIS detection datasets published by Saeys *et al.* (2007), and where these datasets were later used by Chen *et al.* (2014) and Zuallaert *et al.* (2018). Both datasets consist of pretranscript human DNA sequences containing 203 nucleotides (or base pairs but we will use nt for the shorthand of nucleotide for sake of consistency in the rest of the manuscript), with the canonical TIS ATG located at position 61. Note that both datasets are heavily skewed in terms of labels with 1/25 (CCDS) and 1/4913 (Chromosome-21) positive to negative ratio.

Since the release of the aforementioned CCDS dataset, more genes in the human reference genome were annotated (Schneider *et al.* 2017, Genome Reference Consortium 2019). In order to create an additional dataset set to be used for mutations as well as benchmarking, we use 2000 additional nonreplicating TIS-positive sequences from GRCh38.p13 and combine them with 2000 TIS-negative sequences created with the procedure described in (Saeys *et al.* 2007). Furthermore, in order to evaluate our model on all recently discovered genes, we employ nonoverlapping sequences from the Gencode dataset (Frankish *et al.* 2021). Note that all of the data used in this study consist of canonical TIS (ATG), since the prevalence of noncanonical TIS is comparably rare (Zhang *et al.* 2017). An overview of the datasets used can be found in the Supplementary Section B.1.

The dataset utilized in our research was derived from the work of (Saeys *et al.* 2007), as we consider it to be one of the few datasets that accurately captures the fundamental aspects of the topic under investigation, namely translation initiation. A number of recent research efforts make use of longer sequences that encompass the majority of genes (Clauwaert *et al.* 2021) but our preliminary experiments involving sequences with wider sequence windows have revealed that factors that are extraneous to translation initiation can heavily influence the identification of whether a sequence involves translation initiation. Consequently, the problem shifts from identifying translation initiation to the process of translation or even further down the line: the creation of a functional protein. For instance features such as acceptor, donor, and termination sites which are all integral to the creation of a functional protein but not to translation initiation exclusively can divert the model’s attention from translation initiation to these additional factors (Jaganathan *et al.* 2019, Bepler and Berger 2021).

### 2.2 Notation and preprocessing

The problem we tackle in this study is TIS detection, which is a 2-class classification problem with inputs composed of various permutations of four nt, with N∈{A,C,G,T} and with the permutations having a length of 203. A unique property of these data is the codon starting at position 61, which denotes the start of translation (or pseudo-TIS, in the case of TIS-negative sequences). We assign an index of + 1 to A in ATG at position 61 and we label upstream (UTR) and downstream (coding region) nucleotides with decreasing and increasing numbers:


(1) $$\vec{x}=[\underset{\text{Upstream}}{\underset{︸}{N_{-60}N_{-59}\ldots N_{-2}N_{-1}}}\;\mathrm{ATG}\;\underset{\text{Downstream}}{\underset{︸}{N_{+4}N_{+5}\ldots N_{+132}N_{+143}}}].$$

Assuming the above 2-class setting for which a sequence and its categorical association are defined as $x\in {\left\{A,C,G,T\right\}}^{203}$ and y∈{0,1}, let *g* be a classification function that maps the input sequences onto categorical predictions. In this setting, we define the output obtained by a neural network with parameters *θ* and an input ***x*** as $g(\theta ,x)\in {\left[0,1\right]}^{2}$, where this output denotes the probability of each class (also called the softmax output). If the class with the largest probability corresponds to the label for that sequence (that is, $\arg\max_{t}(g{\left(\theta ,x\right)}_{t})=y$), then this sequence is correctly classified.

We use a standard preprocessing routine for one-hot encoding of genomic data, as described in Zuallaert *et al.* (2017, 2018). This routine converts the genomic representation from strings to vectors as follows: with h∈{[1,0,0,0],[0,1,0,0],[0,0,1,0],[0,0,0,1]} for A, C, G, and T, respectively.


(2) $$x=[\underset{\text{Upstream}}{\underset{︸}{h_{-60}\ldots h_{-1}}}\left[\begin{array}{c} 1\\ 0\\ 0\\ 0 \end{array}\right]\left[\begin{array}{c} 0\\ 1\\ 0\\ 0 \end{array}\right]\left[\begin{array}{c} 0\\ 0\\ 0\\ 1 \end{array}\right]\underset{\text{Downstream}}{\underset{︸}{h_{+4}\ldots h_{+143}}}],$$

We also employ nucleotide masking as an augmentation technique in order to enrich the learnt features and to prevent overfitting. The nucleotide masking operation selects a nucleotide at position *m* and replaces the one-hot encoded nucleotide by the zero vector (O=[0,0,0,0]) of the same shape as follows: $\mathrm{mask}(x,m)=\{x_{N_{m}}\to x_{O}\}$.

### 2.3 Deep neural networks

Given enough computational power and data, DNNs have been the go-to models to solve problems related to sequencing in recent years. Prominent models for DNN-based TIS detection include TITER (Zhang *et al.* 2017), TISRover (Zuallaert *et al.* 2018), NeuroTIS (Wei *et al.* 2021b), and DeepTIS (Wei *et al.* 2021a). Among those, NeuroTIS and DeepTIS explicitly incorporate known features that are biologically relevant into the models to enhance accuracy, such as open reading frame and coding region information. This incorporation of known features defeats the purpose of DNNs, which are designed to avoid manual feature extraction. Therefore, we avoid the use of the two aforementioned models. Although the difference in TIS identification effectiveness between TITER and TISRover is not significant, we employ TISRover for our experiments thanks to its elegant design and its slightly superior results.

TISRover is a carefully created convolutional neural network that is specifically created for the TIS detection task described in Section 2.1. We make small modifications to this architecture according to the best-practices of DNN architecture design, incorporate nt masking as an augmentation, and improve the training routine with recent discoveries. Further details on this topic can be found in the Supplementary Section A.

### 2.4 Mutations

Ever since the work of Ingram (1956), mutations in the human exome (i.e. the part of the genome that consists of coding sequences for proteins) have been associated with genetic disorders (Tan 2020). Although mutations originate at the genomic level, they induce negative effects at the protein level, which led early research efforts on the topic of genetic mutations to mainly focus on amino acid altering mutations in the coding regions (Yang *et al.* 2013). However, recently obtained results indicate that mutations in the noncoding regions may easily affect regulatory processes and lead to harmful outcomes too (Scacheri and Scacheri 2015, Zhang and Lupski 2015). Furthermore, noncoding mutations in introns also affect splicing and thus the generated proteins (Jung *et al.* 2021). Not only that, silent mutations (which cause no change in an amino acid sequence) and which were thought to be mostly harmless, were also discovered to play significant roles in cancer prognosis (Gutman *et al.* 2021, Odemis *et al.* 2022). As a result, most of which was thought to be correct with regards to mutations in the past century is currently being re-evaluated.

Mutations in the coding sequence of a genome can roughly be grouped into the following categories:

- **Silent—**If a mutation does not cause a change in the amino acid that is encoded by a particular codon (e.g. CCA to CCG mutation, both encoding glycine) then such a mutation is called a silent mutation.
- **Mis-sense—**When a mutation causes a change in the amino acid encoded by the codon, it is called a mis-sense mutation. For example, a single mis-sense mutation of GAG to GTG in Beta-globin (HBB) causes valine to be synthesized instead of glutamic acid, which causes sickle-cell anemia (Ingram 1956).
- **Non-sense—**When a mutation in a genome results in a premature stop codon (i.e. TAA, TGA, or TAG) in the coding region, which may prevent complete translation of a specific protein, then it is called a non-sense mutation.
- **Noncoding—**Apart from the mutations listed above which occur in the coding region, mutations that occur in noncoding regions of genome are called noncoding mutations.

In order to leverage point mutations, we devise a computational methodology where we mutate, one at a time, a single nucleotide in the given sequence and create additional (mutated) sequences. We provide a formal description of this approach below.

***In silico* point mutations—**Given a sequence ***x***, we define three possible point mutations occurring at a single position *m* as


(3) $$\mathrm{mut}(x,m)=\{x_{N_{m}}\to x_{\{A,C,G,T\}\setminus N_{m}}\}.$$

Sequences in the datasets described in Section 2.1 consist of 203 nucleotides. For sequences of this type, we define all possible point mutations to a single sequence as: where this mutation procedure creates 600 new sequences by transforming a single nt at each position, one at a time, into the other three for 200 positions (not mutating the ATG codon starting at the position 61). This approach, together with the data we employ, enables an *in silico* investigation of the effects of different types of mutations within both the coding and the noncoding regions of a genome.


(4) $$\mu (x)={\left\{\mathrm{mut}(x,m)\right\}}_{m\in \{1,\ldots ,203\}\setminus \{61,62,63\}},$$

Sequences that are of interest to us are the ones that have their predictions changed from TIS positive to TIS negative (i.e. $\arg\max_{t}(g{\left(\theta ,x\right)}_{t})\neq \arg\max_{t}(g{\left(\theta ,x^{\left(m\right)}\right)}_{t})$). Assuming that our model acts as a proxy for the ribosome that initiates the translation process, this means that, due to the introduced mutation, a genomic sequence that would otherwise start the elongation process (i.e. begin synthesizing the protein) will have its gene expression at the level of translation reduced dramatically or fail to synthesize the protein. Given the initial 2000 TIS-positive sequences in Human Genome-M, employing the procedure described above yields 1.2 million unique mutated sequences to conduct a comprehensive analysis of the impact of mutations on translation.

## 3 Results

### 3.1 Models

In order to conduct a thorough investigation, we first aim at designing and training a DNN that achieves state-of-the-art results. As mentioned in Section 2.3, we employ the base architecture of TISRover and denote our modified architecture as TISRover+, training it with the CCDS dataset. Saeys *et al.* (2007) argues that commonly used metrics such as accuracy, true positive rate (TPR), and false-positive rate (FPR) can be misleading for datasets with an extreme skew in labels and propose the usage of FPR at a fixed sensitivity of 0.8 (FPR.80) for benchmarking the Chromosome-21 dataset. For easy comparison with the previous research efforts, we also use FPR.80 as an error measurement on Chromosome-21 and report the effectiveness of different in Table 1.

**Table 1.** FPR.80 effectiveness of the models used in this study on the Chromosome-21 dataset, compared to the literature.

Model	FPR.80
Saeys *et al.* (2007)	0.125
TISRover (reported in Zuallaert *et al.* 2017)	0.031
TISRover (reproduced)	0.032
TISRover+ (improved architecture)	0.030
TISRover+ (improved training routine)	0.028
TISRover+ (randomized nt masking)	0.027
TISRover+ (warm-up)	0.025

As demonstrated, each incremental update applied to the reproduced TISRover model slightly increases the effectiveness of the initial model, even though the margin of improvement left behind by the original model is relatively small. On Chromosome-21, our final model achieves an FPR.80 of .025, thus obtaining state-of-the-art results on this dataset, without providing any explicit guidance to the model in terms of prior biological knowledge. Additional results on the newly curated Human Genome-M as well as Gencode datasets can be found in the Supplementary Section B.

Upon a detailed inspection of predictions made by our final model, we observe that a large portion of TIS-positive and TIS-negative sequences are correctly classified with high confidence into their respective categories while misclassifications are made with relatively lower confidence levels. Not only that, we also observe that our final model is well calibrated in terms of standard calibration error measurements. This means that our model is confident in the majority of the predictions it makes and that for a mutation to change the prediction of a TIS-positive sequence, the mutation under consideration has to cause a strong disruption with regards to TIS identification. In light of these observations, we proceed with the experiments involving mutations.

### 3.2 Mutations

The Human Genome-M dataset described in Section 2.1 contains 2000 TIS-positive sequences that were left out of training in order to perform mutations on these sequences. Nonetheless, not all sequences in this dataset are suitable for experiments that involve mutations due to (i) misclassifications and (ii) unstable sequences that change prediction even with nucleotide masking (before mutations). In order to maximize the trustworthiness of the forthcoming experiments, we filter out the aforementioned sequences. More details about this filtering operation can be found in the Supplementary Section C. The above filtering operation results in 1495 TIS-positive sequences to work with and from these sequences, we create 897 000 mutated sequences using Equation (4). Of those, we observe that 16 148 mutated sequences had their predictions changed due to the introduction of a single mutation (corresponding to 17% of all mutated sequences).

The aforementioned 16 148 mutated sequences that are classified as TIS-negative sequences originate from 1431 unique TIS-positive sequences. This means that, according to our model, 1431 of 1495 TIS-positive sequences would see a failure in translation due to the introduction of a point mutation. In what follows, we examine these 16 148 mutated sequences, as well as the introduced mutations, in more detail in order to understand their biological relevance.

In Table 2, we provide the number of sequences for each mutation type and the proportionality of mutations for each region. Note that the upstream to downstream nt ratio in the dataset we are using is 3/7, which gives mutations occurring in the downstream region more options in terms of different positions to mutate. Nevertheless, we observe that the number of non-sense mutations is disproportionallly large when comparing to the other two types of mutations: 86% of all mutations that affect translation are identified as non-sense mutations occurring in the coding region. In contrast, mis-sense and silent mutations only account for a small fraction (less than 4%). Lastly, noncoding mutations in the 5′-UTR account for approximately 10% of the total.

**Table 2.** Number (proportion) of sequences that had their prediction changed to TIS-negative, considering mutation and region type.

Region	All	Silent	Mis-sense	Non-sense
Anywhere	16 148 (100%)	101 (1%)	412 (2%)	14 001 (87%)
Noncoding	1634 (10%)	0 (0%)	0 (0%)	0 (0%)
Coding	14 514 (90%)	101 (1%)	412 (2%)	14 001 (87%)

**Figure 1.**
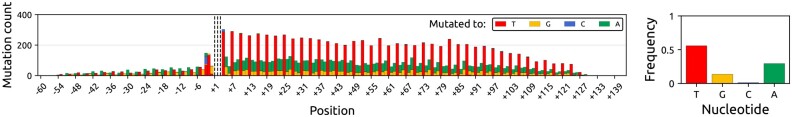
Histogram of nucleotides that cause a mutation that leads to a TIS-negative prediction, and their proportionality.

**Figure 2.**
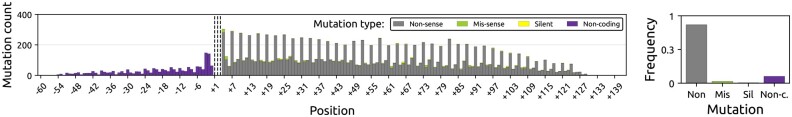
Histogram of nonsense mutations, mis-sense mutations, and silent mutations, and their proportionality.

To give an overall view on the mutations and their distribution for each position, we present Fig. 1 which shows point mutations that caused a change in prediction. Following that, in Fig. 2, we provide the types of mutations for each position. We observe that neither the positions nor the nucleotides of the mutations are uniformly distributed. Interestingly, we can observe that the mutations that affect translation occur less frequently towards the 3′ end, and we can also observe that mutations in the final 11 positions amount to none. While T mutations account for almost half of all mutations, C mutations only account for less than 1%.

## 4 Discussion

In what follows, we provide further insights into the biological relevance of the observations presented in Section 3.

### 4.1 Neural networks learn biologically relevant features

#### 4.1.1 Integrity of Kozak sequence

**Observation—**Our experiments indicate that mutations made at position −3, particularly the ones that mutate purines into pyrimidines, have a significant impact on translation, with this mutation being the most influential one over other mutations in the 5′-UTR (see Fig. 1). Apart from position −3, mutations introduced at position −2 (into T) and −1 (into G) are the second and the third most impactful ones in the 5′-UTR. When investigating the coding region, we can also observe that mutations made at position +4 have the largest impact on translation initiation.

**Biological significance—**In eukaryotes, cap-dependent translation initiation begins when the start codon, with the help of eIFs, is accommodated at the ribosome. For higher eukaryotes, the mRNA sequence in the vicinity of the start codon, which is called the Kozak sequence, plays an important role in this process (Simonetti *et al.* 2020). This sequence is often defined as 5′-GCCRCCATGG-3′, with R representing a purine (i.e. A or G) and ATG representing the start codon (Simonetti *et al.* 2020). In this sequence, while the integrity of nt in other positions are significant, the nt most influential to translation initiation are the ones residing at positions −3 (R) and +4 (G) relative to the start codon. As a result, Kozak (1987) observed that mutations occurring at positions −3 and +4 have the strongest influence in preventing translation, and these observations align with our experimental observations.

#### 4.1.2 uATG mutations in 5′-UTR

**Figure 3.**
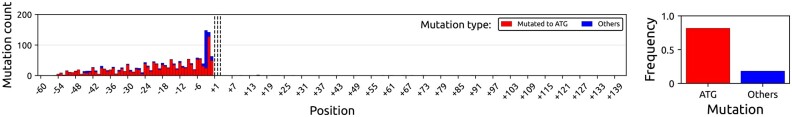
Comparison of ATG mutations in the 5′-UTR to other mutations, and their proportionality. In the left figure, the *y*-axis is scaled between 0 and 200 for better visibility.

**Observation—**Delving deeper into the mutations in the 5′-UTR, we observe that our model estimates that ATG mutations in the 5′-UTR (uATG) would lead to significant reduction in translation initiation likelihood, with uATG mutations accounting for the majority of the mutations in the upstream region. In Fig. 3, we show the number of mutations that result in an uATG triplet and its comparison to remaining noncoding mutations. Note that the ATG mutated triplets only lead to a prediction change in the 5′-UTR.

**Biological significance—**Due to the numerous problems caused by ATG mutations in the 5′-UTR, it is generally believed that the presence of uATG mutations of eukaryotic genomes decreases mRNA translation efficiency (Rogozin *et al.* 2001). One such problem is the creation of an uATG codon close to the original TIS in the open reading frame (ORF), altering the initiation point and possibly resulting in an elongated peptide (Semler *et al.* 2012, Lazarus *et al.* 2014). Another, more severe problem is the potential creation of an uATG codon in a different reading frame than the ORF. In such cases, a completely different protein is synthesized due to the frame shift, potentially leading to genetic disorders (Damjanovich *et al.* 2011). ATG triplets in nonoptimal sequences can also lead to leaky scanning (Wang and Rothnagel 2004).

Although mutations in the coding region were the main focus of past research efforts, recent results indicate that many genetic diseases originate from mutations in the 5′-UTR, with these mutations causing problems in protein synthesis. Supporting the recent findings of Zhang and Lupski (2015) and Steri *et al.* (2018), our model suggests that approximately 10% of the mutations that have a significant impact on translation are noncoding mutations in the 5′-UTR. These findings are complementary to those of (Abad-Navarro *et al.* 2018), who studied the effect of mutations to the start codon. While most such mutations lead to the loss of translation, a small fraction turns out to be benign, for example due to the presence of a start codon in the coding sequence, resulting in the production of a shortened but functionally equivalent protein.

#### 4.1.3 Premature stop codons in the coding region

**Observation—**Investigating the coding region that will determine the amino acid sequence, Fig. 2 shows that almost all of the detrimental mutations in the coding region are non-sense mutations that lead to the creation of a stop codon. (Note that a small portion of the mutations are mis-sense mutations, but we observe them at positions +4 and +5, which makes them more relevant to the Kozak sequence rather than the downstream region.) Not only do the non-sense mutations make up almost all of the mutations in the coding region (96%), they also account for most of the mutations when the 5′-UTR mutations are accounted for (86%). As such, our model indicates that non-sense mutations that appear shortly after the initiation of translation are extremely impactful for protein synthesis.

**Biological significance—**The final stage of translation of an mRNA molecule is termination, which signifies the end of the elongation of the protein. For protein synthesis, accurate termination of elongation by release factors is crucial for the integrity of cellular proteomes (Svidritskiy *et al.* 2018). Release factors perform the termination by identifying stop codons (TAA, TGA, TAG). A premature termination of translation may lead to the accumulation of truncated proteins, which are detrimental to the cell (Johnson *et al.* 2011). However, more often than not, a premature termination leads to translational errors due to unstable mRNA, and where these faulty mRNAs go through a process known as nonsense-mediated mRNA decay, a specialized mechanism for rapid degradation of faulty mRNAs (Amrani *et al.* 2006). To add, many genetic diseases are known to originate from non-sense mutations in coding regions, which indicates that errors that occur at the translational level are extremely costly to human health (Sicinski *et al.* 1989, Pinotti *et al.* 2006).

Mutations that lead to premature stop codons are problematic because they terminate the translation process before a functional protein has been made. We observe that the number of the aforementioned problematic mutations lessens as we move further into the downstream area (see Fig. 2). We further observe that the final 20 positions only have a handful of prediction-altering mutations, with the last 14 positions not having such mutations at all. The lack of problematic mutations in this region can likely be attributed to the creation of a functioning, near-complete protein, which stops the forthcoming stop codons from preventing translation initiation (i.e. these stop codons end the process of translation gracefully).

Note that the first nt of each codon in the downstream region has more representation for mutations compared to others. This is because of the difference in frequency of codons that can readily be mutated to stop codons. We invite interested readers to the Supplementary Section D for an extensive analysis on this topic.

#### 4.1.4 Premature stop codons in the other reading frames

**Figure 4.**
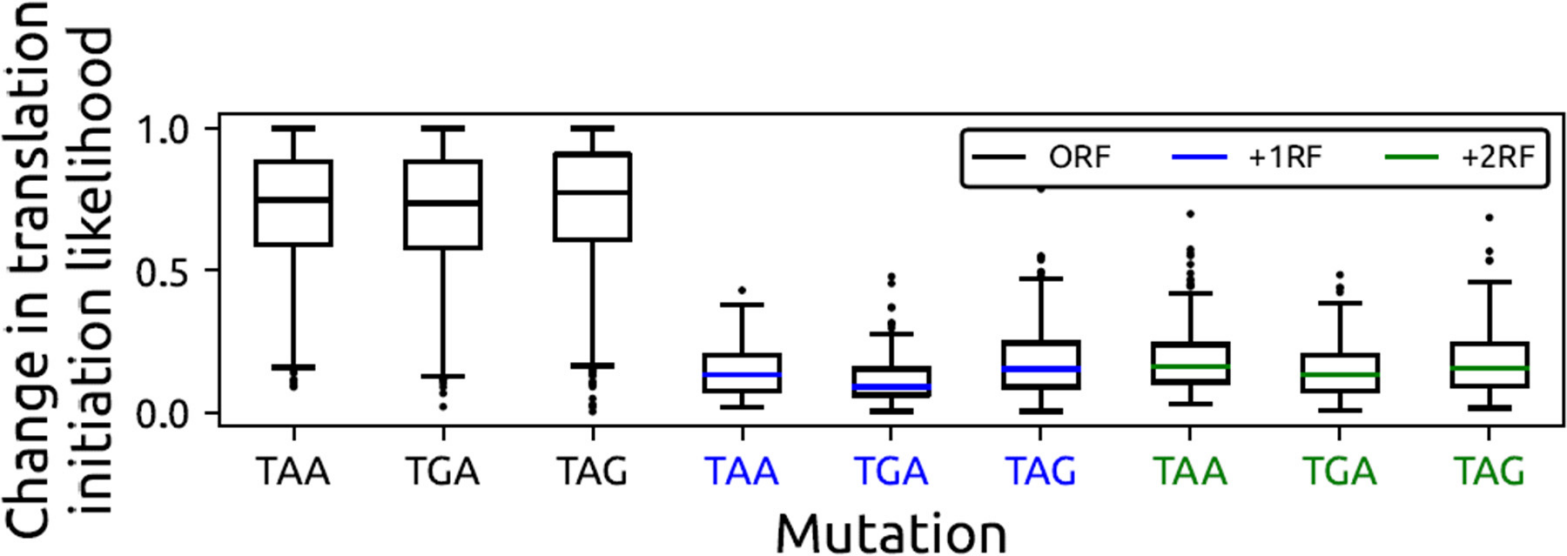
Change in the translation likelihood for mutations that create a stop codon in the three different reading frames.

**Observation—**We investigate the change in the translation likelihood observed from our model when accounting for stop codons in the ORF and in the other reading frames. In Fig. 4, we show box plots of the changes in prediction likelihood for the three stop codons that occur in the three reading frames. These results indicate that our model believes that premature stop codons are only detrimental to translation when they occur in the ORF and not in the other reading frames.

**Biological significance—**For a stop codon to signify the termination of translation, it needs to exist in the ORF of an exon, which is what we observe in Fig. 4.

#### 4.1.5 Insignificance of cytosine mutations to translation

**Observation—** Fig. 1 indicates that C mutations account for less than 1% of the total number of mutations that cause problems for translation, as demonstrated by our model.

**Biological significance—**Since cytosine may go through spontaneous deamination due to heat or ultraviolet light, C to T (or C to U) mutations are the most commonly occurring mutations in the human genome. In an attempt to prevent problems in protein synthesis that may arise due to this spontaneous mutation, it is hypothesized that the evolutionary process detached C in DNA from most of the regulatory processes regarding translation (Wang 2020). Indeed, none of the stop codons, the canonical start codon, or two of the most commonly observed noncanonical start codons contain C.

One of the well-known gene structures that is related to transcription (and thus to translation) are CG islands, where these islands are located around the promoters of transcription. However, in an attempt to minimize potential issues that may arise in these regions, cells quickly identify C to U mutations and repair them with a process called CG suppression, thus minimizing the impact of C mutations.

### 4.2 Case study for Beta-globin gene

An infamous case of point mutations leading to detrimental outcomes in humans is the family of Thalassemia disorders arising due to mutations in the globin genes (Thein 2013). A subset of Thalassemia disorders called Beta thalassemia arise due to certain mutations in the Beta-globin gene where these mutations reduce beta chains in hemoglobin which then cause severe anemia. Estimations in 2015 show that approximately 280 million people suffer from a Thalassemia variant (Vos *et al.* 2015) caused by one of the 300 known mutations, with approximately 450 thousand people having severe cases. In the same year, approximately 17 000 deaths were attributed to Thalassemia.

To confirm the correctness of the overall observations made in Section 4.1, we delve deeper into Beta-globin gene and investigate a number of point mutations that cause Beta thalassemia. We show that our model is able to identify some of the well-documented pathological mutations described in the work of (Thein 2013). In particular, our model finds that a number of frame shift mutations as well as nonsense mutations in the coding region directly influence translation initiation since those mutations lead to the creation of another open reading frame.

Apart from the pathological mutations mentioned above, we also investigate 34 benign mutations that are documented in the work of (Sherry *et al.* 2001). Since these mutations are benign, translation occurs without any problems. Therefore, our primary objective in examining benign mutations using our model is to confirm its proficiency from another angle: these mutations should not influence the prediction of the model on the HBB gene. Indeed, experiments using our model indicate these mutations to be not influential for translation.

Taking the these experiments a step further, we investigate all point mutations for 200 nt in the Beta-globin gene (similar to the experiments conducted in Section 3) and identify 12 mutations to be potentially problematic, of which 5 are documented and 7 are thus far undocumented in the literature. Of those 7 undocumented mutations we conjecture a single one (HBB + 25 A →T) to be the most detrimental one due to its striking effect on the translation likelihood obtained by our model.

Note that the observations made above are done so with a single model that achieved the best performance across others on the evaluated datasets. In order to evaluate the confidence of our observations, we train 50 additional models with bootstrapping and re-evaluate the HBB mutations described above. Our results indicate that the majority of our critical observations with regards to HBB mutations come with high confidence.

Detailed discussions on this topic as well as experimental results are presented in the Supplementary Section E.

### 4.3 Extracting novel observations from neural networks

#### 4.3.1 Which mutations are more detrimental to translation?

**Figure 5.**
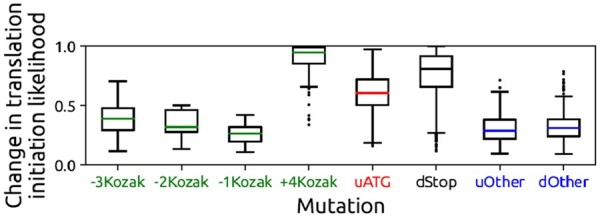
Change in the translation likelihood for various mutations that fall into the categories discussed in Section 4.1.

Measuring the impact of mutations on the process of translation is not straightforward. However, we can use the magnitude of prediction changes as a metric for identifying the impact of mutations on translation. Doing so, through box plots shown in Fig. 5, we present the change in the translation likelihood after the mutation takes place, for the various mutations discussed in Section 4.1. We observe that mutations at the +4 position (+4Kozak), as well as ones that create stop codons in the early downstream area (dStop), are the most impactful ones, compared to other mutations. Indeed, these mutations are known to prevent the translation completely, instead of just reducing gene expression levels. uATG mutations closely follow the above two, followed by other mutations in the rest of the Kozak sequence. Finally, we group the remaining mutations in other regions for upstream (uOther) and downstream (dOther) separately. We invite interested readers to the Supplementary Section F for additional details on this topic.

#### 4.3.2 Detrimental non-ATG mutations in the 5′-UTR

While mutations in the 5′-UTR were believed to be mostly harmless in the past and were mostly ignored (compared to mutations in the coding region), research efforts that took place in the past decade indicate that silent mutations could have harmful effects on mRNA stability (Landi *et al.* 2012) and on most, if not all, phases of the gene expression process, leading to a reduction or an increase in protein quantities (Diederichs *et al.* 2016, Gutman *et al.* 2021). Since this discovery regarding mutations in noncoding regions is gaining research interest in recent years, we now describe our findings in this area.

In Section 4.1, we covered the biological relevance of a large number of mutations that were identified by our model to be detrimental to translation. Of the 16 148 mutations that our model determined to be harmful, 149 mutations correspond to non-ATG mutations in the 5′-UTR. As shown in Fig. 5, these mutations (grouped into uOther) are identified to be as detrimental as some of the mutations that occur in the Kozak sequence. In order to put these findings into perspective, we would like to bring attention to the number of mutations that were performed. According to the experimental routine described in Section 2.4, of 879 000 mutations, 358 000 were performed in the 5′-UTR, and of those 358 000 mutations, our model identified a select few (149) to be detrimental to translation in humans. We do not yet know why these mutations are deemed harmful to translation by our model but we conjecture that these findings may indicate the existence of rare and undiscovered regulatory elements in the 5′-UTR for translation which get disrupted by mutations (Tautz 2009). Interestingly, we also observe the existence of sequences which have their predictions changed due to multiple non-uATG mutations in the 5′-UTR. We believe that those cases may signify the existence of undiscovered elements related to binding patterns of certain initiation factors. In the Supplementary Section G, we provide detailed information about these sequences, as well as the frequency of the aforementioned mutations that disrupt translation.

## 5 Conclusions and future research

By employing state-of-the-art training routines, we put together a DNN that outperforms already existing approaches for TIS detection in human genomic data. Through the usage of *in silico* point mutations, we then demonstrated that our DNN learns a number of well-defined signals that are biologically meaningful. Taking these observations one step further, and with the goal of guiding future research efforts, we documented a number of observations of interest regarding (i) harmful mutations to the Beta-globin gene, (ii) the severity of the mutations on translation, and (iii) the presence of rare mutations in the 5′-UTR that disrupt translation. A highly relevant and underexplored direction for future research is the exploration of other types of mutations such as frame shifts, deletions, and insertions as well as combinatory point mutations.

Getting DNNs to describe the biological relevance of what was learned from the data and extracting novel biological knowledge from DNNs are two tasks that are not easy to accomplish. In this research effort, we demonstrated the usefulness of *in silico* mutations, in combination with meticulous experimental routines, to achieve a certain degree of biological relevance and to obtain novel insights into translation. However, this does not mean that the used approach is the only way to capture such insights. On the contrary, we encourage researchers in this line of work to devise novel and innovative biologically relevant experiments to get DNNs to describe their predictions and to extract biologically meaningful insights.

As a cautionary remark, we note that in isolation, the utility of observations generated by neural networks can be limited. To enhance the reliability and applicability of our findings, we have incorporated mutations observed in the population such as documented mutations for the Beta-globin gene. Moving forward, in scenarios when they are relevant, we recommend that researchers continue to leverage mutation databases as well as biological tools such as SIFT (Ng and Henikoff 2003), Polyphen2 (Adzhubei *et al.* 2010), and CADD (Rentzsch *et al.* 2019)—which quantify the impact of mutations through the use of a multiple sequence alignment—to confirm research outcomes from multiple angles.

## Supplementary Material

btad338_Supplementary_Data

## Data Availability

Datasets, the final model (TISRover+), detailed mutation results, and code snippets for model evaluation can be found in github.com/utkuozbulak/mutate-and-observe.
