# Supplementary material for: Mutate and observe: utilizing deep neural networks to investigate the impact of mutations on translation initiation
Source: Bioinformatics. 2023 May 24;39(6):btad338. doi: 10.1093/bioinformatics/btad338 (PMC10250082; doi:10.1093/bioinformatics/btad338)
Supplement: btad338_Supplementary_Data [file btad338_supplementary_data.pdf]

Supplementary Material for: Mutate and Observe: Utilizing Deep Neural Networks to Investigate the Impact of Mutations on Translation Initiation

A Deep Neural Networks and Training

As described in the main text, we use TISRover, a carefully designed DNN for the task of TIS detection (see Figure 1) that is composed of four convolutional layers followed by a linear one. Our primary experiments on this model with the dataset described in the paper revealed that this model is able to overfit the training data very quickly which leads to subpar results on test and validation datasets. In an attempt to overcome this challenge as well as to allow experimentation with mutations, we distill the original model into a more lightweight one while incorporating base pair (bp) masking as a method of data augmentation. We detail our modifications to the original architecture and training methodology as follows:

- **Convolutional kernels** – Keeping the number of convolutional layers the same as the TISRover architecture, we reduce the number of filters to the closest smaller number that is a power of 2. As a result, we reduce the filter counts from 70 to 32 in the first, 100 to 64 in the second, and finally 150 to 128 in the third convolutional layer. We do not modify the convolutional kernels as we have observed the setup to be optimal for the data at hand. The reduction in the number of filters in the convolutional layers correspond to a reduction of $36,440$ trainable parameters, which approximately corresponds to half the trainable parameters of convolutional layers in TISRover.
- **Dropout positioning** – We follow [10] and [9] and remove the dropout layers during the convolution phase of the model and instead apply a dropout of frequency (0.5) only once before the final linear layer, similar to [9].
- **Linear layers** – We experiment with one, two, and three layers of linear layers (also called dense or fully-connected layers) after the flattening of features after convolutions. We find that the two layer setup yields the best results and do not change it compared to TISRover. However, we modify the number of neurons to be in line with the reductions in the number of convolutional filters. This operation reduces the number of trainable parameters in linear layers from $462,338$ to $98,690$, corresponding to a reduction of 80%.
- **Warm-up** – Linear warmup training has proven to be beneficial for training in recent research efforts. We employ this technique to perform a single epoch of warmup where the learning rate starts from 0 and linearly increases to the initial learning rate at each batch.
- **Optimizer** – We continue to use SGD as an optimizer and train our model for 50 epochs but set the initial learning rate to 0.1 instead of 0.05 as in TISRover. Furthermore, following [5], instead of decaying the learning rate with multiplier of 0.5 for each 10 epochs, we decay it with a multiplier of 0.1 for every 20 epochs.
- **Class weights** – We also experiment with class weights as a method to combat the data imbalance (1 to 26 positive to negative ratio) and find that the usage of class weights positively affects the training outcome (by small margins). Although the class weights do not significantly affect the results, we find that weighting positive sequences 4 times as a single negative one yields the best results.

- **Batch size** – Although we experimented with batch sizes of range $\{32, 64, 128, 256\}$ (with accordingly adjusted learning rates), we found that the batch size of 32 leads to the best results, similar to the original TISRover architecture.
- **Masking** – In an attempt to overcome the data imbalance as well as to accommodate for the subsequent experiments involving mutations, we employed base pair (bp) masking as a method of data augmentation. Given a sequence $\boldsymbol{x}$ composed of one-hot representations, we replace the bp at the position m with a zero vector $\mathbb{0} = [0, 0, 0, 0]$ as follows:

$$\texttt{mask}(\boldsymbol{x}, m) = \{\boldsymbol{x}_{\texttt{N}_m} \rightarrow \boldsymbol{x}_{\mathbb{0}}\} \,. \tag{1}$$

  We evaluate a number of different scenarios for bp masking and find randomized masking up to a certain number (in this case, 40) to be the one that improves the results the most. As such, during training, for each sequence we sample a random number $k \in [0, 40]$ and mask the sequence at k different positions. Naturally, when $k = 0$, then no bp is masked and the sequence is fed to the network as-is.

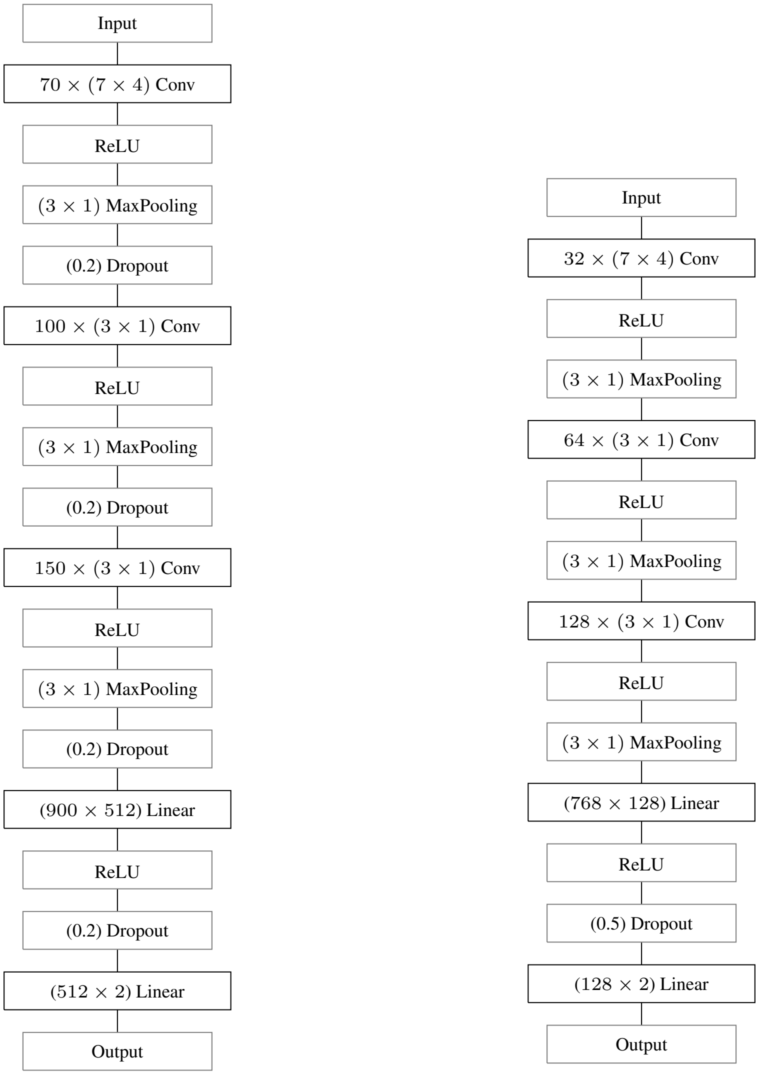

Architecture: TISRover
Trainable parameters: $530,618$

Architecture: TISRover+
Trainable parameters: $130,530$

Figure 1: Detailed descriptions for TISRover and TISRover+ architectures.

B Additional Details on Experiments

B.1 Datasets and model performance

In Table 1, we provide additional details about the properties of datasets used in this work.

Human-Genome-M – Performance of our models on the newly created `Human Genome-M` dataset can be found in Table 2. As it can be seen, on the newly curated `Human Genome-M` dataset, our final model achieves an overall accuracy of .916 (.86 TPR and .98 FPR) when adopting standard accuracy evaluation using $\arg\max(g(\theta, \boldsymbol{x})) \geq .5$. When using the best threshold calculated with an ROC curve, the accuracy further increases to .946 (.95 TPR and .94 FPR).

Gencode – In order to maintain comparability with past research efforts, we used the dataset published in the work of [6] which was published in 2007. Naturally, this dataset lacks the most recent genomic sequences. In order to demonstrate the effectiveness of our model and to addresss this potential limitation, we employed the latest GENCODE dataset (GENCODE release 43 corresponding to GRCh38.p13), an extensive reference annotation resource for the human genome. In particular, we employed protein-coding transcript sequences from the most recent reference chromosomes of humans and reformatted the dataset by trimming the sequences to 203 nucleotides and relocating the start codon (ATG) to position 61, conforming to the input requirements of our model. Then, we remove all overlapping sequences between this dataset and our previous datasets (`CCDS`, `Chromosome-21`, `Human Genome-M`) which leaves us with a total of 73,590 novel sequences. We use this dataset to evaluate our models without any additional training to observe whether our models can identify most-recently discovered transcription sequences.

In Table 3, we provide results on the `GENCODE` dataset with our models that are incrementally improved. Note that the `GENCODE` dataset contains only TIS-positive samples obtained from human genome, thus, the accuracy corresponds to ACC $=$ TP$/$FN. We also experiment with the thresholding where this threshold is calculated based on the `Human Genome-M` dataset and find that our final model can successfully identify 92.3% (without thresholding) and 98.5% (with thresholding) of all sequences in the `GENCODE` dataset as correctly belonging to the TIS-positive class, indicating the usefulness of features learned by the model.

B.2 Generalization of Experimental Results

The bootstrap method (see [1]) is a resampling technique that has been widely used in statistical analysis to estimate the uncertainty associated with a given estimate or model. It is a non-parametric approach that involves repeatedly resampling a dataset with replacement, and using the resulting samples to estimate a statistic of interest. One of the key advantages of this method is that it does not require any assumptions about the distribution of the data, which makes it useful in situations where traditional statistical methods may not be appropriate or too complex. This is especially relevant

Dataset	Total	TIS-positive sequences	TIS-negative sequences	Pos./Neg. ratio	Source
`CCDS`	364,495	13,917	350,578	0.0396	[6]
`Chromosome-21`	1,267,701	258	1,267,443	0.0002	[6]
`Human Genome-M (GRCh38.p13)`	4,000	2,000	2,000	1	[7]
`Gencode (GRCh38.p13)`	73,590	73,590	0	-	[2]

Table 1: Characteristics of datasets used in this study.

Model	Accuracy	TPR	FPR	FPR.80
TISRover+ (improved architecture)	.806	.630	.996	.015
TISRover+ (improved training routine)	.831	.692	.985	.024
TISRover+ (randomized nt masking)	.858	.723	.994	.016
TISRover+ (warm-up)	.916	.861	.987	.009
TISRover+ (confidence thresholding)	.946	.959	.949	.009

Table 2: Overall effectiveness of the models used in this study on the `Human Genome-M` dataset.

Model	Accuracy	Accuracy with threshold
TISRover+ (improved architecture)	0.913	0.962
TISRover+ (improved training routine)	0.921	0.968
TISRover+ (randomized nt masking)	0.919	0.963
TISRover+ (warm-up)	0.923	0.985

Table 3: Accuracy of traned models on the `GENCODE` dataset. The threshold for the third column is calculated from the `Human Genome-M` dataset.

in the context of machine learning, where the data is often high-dimensional and the underlying distribution is unknown or non-normal.

In order to assess the stability of our model and its generalization performance, which is crucial when dealing with real-world data, we employ bootstrapping technique and train 50 additional models. The performance of the bootstrapped models is described in Table 4 and is generally comparable (though somewhat lower) with the performance of the model that was trained on the full dataset. This indicates that our model has good generalization performance and is likely to do well on new data.

Note that the mean bootstrap accuracies are indeed somewhat lower than the accuracy of the full model. This is because the bootstrapped models are trained on subsets of the full dataset, obtained by sampling with replacement. Even though there are strategies to approximately correct this negative bias (see [1]), we chose not to employ them, as their appropriateness for deep neural networks may not be assured. Moreover, our conclusion regarding generalization performance is in fact sharpened by showing that the model continues to perform well even under slightly pessimistic assumptions.

B.3 Prediction Confidence and Confidence Calibration

Confidence calibration refers to the ability of machine learning models, such as a neural networks, to output predictions that are well-calibrated with their true probability of being correct. It is a critical aspect of model reliability, as it allows practitioners to assess the quality of model predictions and make informed decisions based on them. Moreover, confidence calibration is vital in detecting and mitigating model errors and biases. A poorly calibrated model may exhibit overconfidence in its predictions, leading to untrustworthy predictions. By ensuring proper calibration, machine learning practitioners can gain insights into the model's behavior and use this knowledge to refine the model and improve its performance.

In order to measure the calibration confidence of the models evaluated in this study, we employ three metrics: negative-log likelihood loss (NLL), estimated calibration error (ECE), and Brier score.

NLL – Negative log loss, also known as cross-entropy loss, is a commonly used metric for evaluating the performance of a classification model. In the context of confidence calibration, negative log loss measures the discrepancy between the predicted probabilities and the true probabilities of the events being classified. Given a probabilistic model $f(Y|X)$, NLL for N data points is defined as:

$$\text{NLL} = -\sum_{n=1}^{N} \log(f(y_i|\boldsymbol{x}_i)) \,. \tag{2}$$

ECE – Estimated calibration error (ECE) is a popular metric for assessing the calibration performance of a classification model. In the context of confidence calibration, ECE measures the difference between the predicted probabilities and the true probabilities of the events being classified, averaged over a set of confidence intervals. Following [4], we calculate ECE as follows:

$$\text{ECE} = \sum_{m=1}^{M} \frac{|B_m|}{n} \left|\text{acc}(B_m) - \text{conf}(B_m)\right| \,, \tag{3}$$

where the error is measured over M bins of confidence intervals with B_m referring to the set of indices of samples whose prediction confidence falls into the interval $(\frac{m-1}{M}, \frac{m}{M}]$.

Brier score – The Brier score is a widely used metric for evaluating the performance of probabilistic predictions generated by a classification model. It is a proper scoring rule that measures the mean squared difference between the predicted probabilities and the true binary outcomes of the events being classified. Given a probabilistic prediction $g(\theta, \boldsymbol{x})$ and its label y, Brier score is calculated as follows:

$$\text{Brier} = \sum_{n=1}^{N} (g(\theta, \boldsymbol{x}_n) - y_n)^2 \,. \tag{4}$$

Results on Confidence Calibration – In Table 5 and Table 6, we provide calibrations of incremental models evaluated in the main text measured with NLL, ECE, and Brier score. Apart from these results, we also experiment with a post-processing routine called temperature scaling [4] to observe the potential shortcomings of the models and whether this postprocessing step leads to dramatic improvements. As it can be seen, all three metrics lead to small calibration errors (often < 0.1), indicating that the models used having desirable properties in terms of calibration.

Prediction Confidence – Apart from the calibration results discussed above, we also present the histogram of prediction confidences for correctly classified TIS-positive and TIS-negative samples as well as incorrect classifications in Figure 2, Figure 3, and Figure 4 for `Chromosome-21`, `Human Genome-M`, and `GENCODE`, respectively. Prediction confidences on the aforementioned datasets are taken from the final model which we performed the mutations on.

As it can be seen, the majority of the TIS-positive and TIS-negative sequences in all three datasets are correctly classified with high confidence. To add, while misclassifications in `Human Genome-M` dataset follow a pattern of uniform distribution, for the other two datasets, misclassifications come with lower confidence values, indicating that the mistakes made by our model are, more often than not, without overconfidence.

Chromosome-21	Human Genome-M			GENCODE
FPR.80	Accuracy	TPR	FPR	Accuracy
0.040	0.886	0.795	0.991	0.839
0.036	0.908	0.856	0.976	0.929
0.035	0.903	0.837	0.985	0.891
0.039	0.865	0.750	0.994	0.799
0.039	0.921	0.878	0.979	0.922
0.036	0.898	0.831	0.981	0.884
0.038	0.905	0.837	0.988	0.900
0.038	0.880	0.780	0.995	0.833
0.039	0.918	0.875	0.978	0.914
0.039	0.882	0.795	0.984	0.858
0.037	0.912	0.863	0.978	0.916
0.033	0.904	0.840	0.983	0.884
0.039	0.863	0.747	0.994	0.801
0.039	0.891	0.805	0.993	0.862
0.037	0.908	0.870	0.962	0.917
0.039	0.876	0.779	0.987	0.842
0.039	0.847	0.713	0.997	0.752
0.031	0.805	0.627	0.998	0.680
0.032	0.908	0.850	0.982	0.891
0.038	0.909	0.854	0.981	0.917
0.038	0.843	0.704	0.996	0.763
0.040	0.910	0.856	0.980	0.912
0.033	0.845	0.712	0.993	0.774
0.036	0.879	0.788	0.984	0.851
0.035	0.905	0.853	0.973	0.909
0.039	0.908	0.863	0.968	0.917
0.039	0.888	0.800	0.991	0.862
0.037	0.916	0.877	0.971	0.919
0.038	0.884	0.797	0.987	0.851
0.030	0.908	0.847	0.985	0.908
0.030	0.784	0.584	0.998	0.642
0.040	0.890	0.812	0.983	0.861
0.039	0.886	0.796	0.992	0.854
0.039	0.900	0.835	0.980	0.891
0.040	0.903	0.856	0.967	0.903
0.036	0.876	0.779	0.987	0.842
0.039	0.902	0.840	0.980	0.894
0.037	0.881	0.802	0.976	0.859
0.036	0.857	0.738	0.992	0.785
0.036	0.865	0.751	0.994	0.802
0.033	0.879	0.783	0.990	0.828
0.040	0.850	0.718	0.997	0.765
0.036	0.899	0.837	0.978	0.894
0.034	0.905	0.850	0.975	0.921
0.031	0.900	0.833	0.983	0.892
0.038	0.891	0.813	0.984	0.859
0.038	0.914	0.883	0.961	0.926
0.041	0.889	0.810	0.982	0.864
0.036	0.859	0.736	0.996	0.788
0.039	0.893	0.817	0.985	0.879
0.0377 ±0.0036	0.8909 ±0.0327	0.7943 ±0.0716	0.9817 ±0.0114	0.8545 ±0.0629

Table 4: Performance of 50 bootstrapped models on evaluated datasets. The last row depicts the mean (± std) performance calculated from all bootstrapped models.

Model	Without additional calibration NLL	ECE	Brier	Temperature scaling NLL	ECE	Brier
TISRover+ (improved architecture)	0.043	0.012	0.011	0.042	0.010	0.011
TISRover+ (improved training routine)	0.042	0.012	0.011	0.040	0.010	0.011
TISRover+ (randomized nt masking)	0.035	0.011	0.009	0.034	0.009	0.009
TISRover+ (warm-up)	0.034	0.003	0.010	0.035	0.006	0.010

Table 5: Calibration properties of trained models on the `Chromosome-21` dataset.

Model	Without additional calibration NLL	ECE	Brier	Temperature scaling NLL	ECE	Brier
TISRover+ (improved architecture)	0.406	0.080	0.120	0.343	0.033	0.111
TISRover+ (improved training routine)	0.361	0.079	0.115	0.320	0.036	0.106
TISRover+ (randomized nt masking)	0.403	0.091	0.126	0.346	0.046	0.115
TISRover+ (warm-up)	0.196	0.012	0.057	0.195	0.012	0.057

Table 6: Calibration properties of trained models on the `Human Genome-M` dataset.

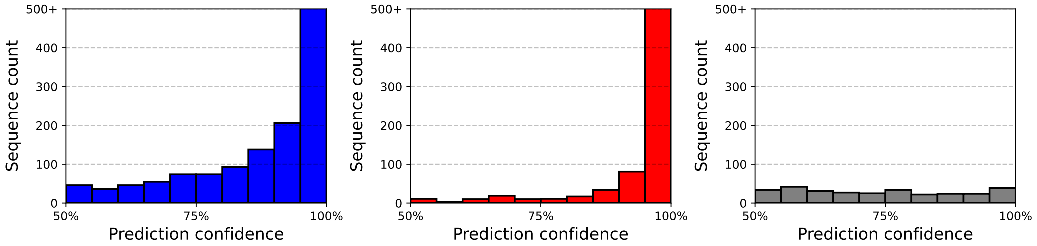

(a) TIS-positive (b) TIS-negative (c) Incorrect classifications

Figure 2: Prediction confidences obtained from the final model on `Chromosome-21` dataset for sequences that are correctly classified as (a) TIS-positive and (b) TIS-negative. Prediction confidences for misclassified TIS-positive and TIS-negative sequences are shown in (c).

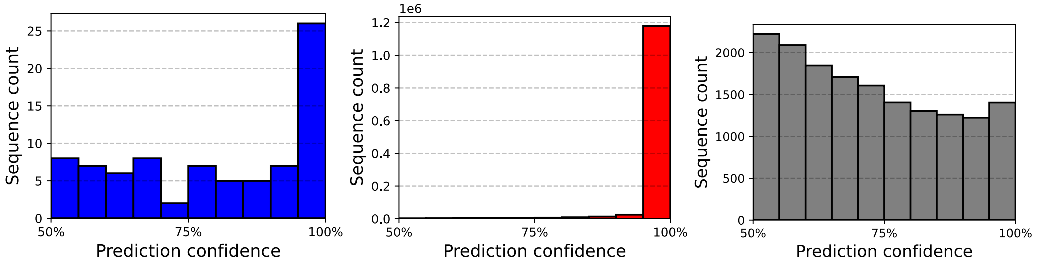

(a) TIS-positive (b) TIS-negative (c) Incorrect classifications

Figure 3: Prediction confidences obtained from the final model on `Human Genome-M` dataset for sequences that are correctly classified as (a) TIS-positive and (b) TIS-negative. For both TIS-positive and TIS-negative sequences, histogram for the confidence of misclassified sequences are given in (c). Note that `Chromosome-21` dataset is extremely imbalanced and contains $1,267,443$ TIS-negative samples while only containing 258 TIS-positive sequences.

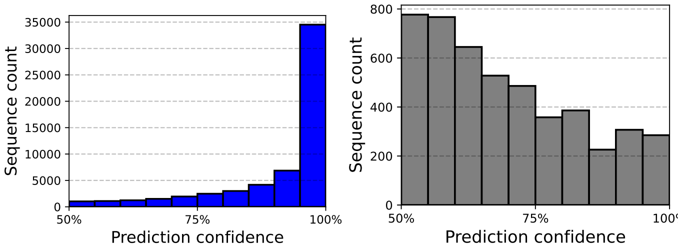

(a) TIS-positive (b) Incorrect classifications

Figure 4: Prediction confidences obtained from the final model on `GENCODE` dataset for sequences that are correctly classified as (a) TIS-positive and (b) TIS-negative. For both TIS-positive and TIS-negative sequences, histogram for the confidence of misclassified sequences are given in (c). Note that the `GENCODE` dataset does not contain any TIS-negative samples, thus the missing figure for corresponding sequences.

C Discarding Unstable Sequences

In the main paper, we indicated that 505 sequences from `Human Genome-M` dataset were not used for experiments involving mutations. Those sequences fall into two categories: (1) incorrectly classified sequences and (2) unstable sequences that are not suitable for mutations. In what follows, we explain how those sequences are discarded.

Discarding incorrectly classified sequences – The `Human Genome-M` dataset described in Section 2.1 of the main text contains 2, 000 TIS-positive sequences that were left out of training in order to perform mutations on. Nonetheless, not all sequences in this dataset are suitable for experiments that involve mutations. Of 2, 000 sequences, 278 are incorrectly classified by the model as belonging to the TIS-negative class, therefore we discard them.

Discarding unstable sequences – In order to maintain the authenticity of experiments involving mutations, we take another step in identifying sequences that are not suitable by investigating the validity of predictions for sequences when bps are masked one at a time. Our approach is quite straightforward: similar to mutations, we mask, one at a time, a single bp in the given sequence and create additional masked sequences (200 masked sequences for each sequence) using Eq 3 in the main paper. If any of the 200 masked sequences have their predictions changed, we discard the (unmasked) sequence. Our rationale for this operation is simple: these sequences are already misclassified due to the masking of a bp, before any mutations take place. As such, including them in experiments involving mutations may result in misleading observations when it comes to investigating the nature of mutations. Doing so, we get rid of 227 additional sequences. In Figure 5 we provide an example for the discarding operation using masks detailed above.

`Original :`	CCGGC $\rightarrow$	Positive	`Original :`	TGGAC $\rightarrow$	Positive
`Mask pos1:`	-CGGC $\rightarrow$	Positive	`Mask pos1:`	-GGAC $\rightarrow$	Negative
`Mask pos2:`	C-GGC $\rightarrow$	Positive	`Mask pos1:`	T-GAC $\rightarrow$	Positive
`Mask pos3:`	CC-GC $\rightarrow$	Positive	`Mask pos1:`	TG-AC $\rightarrow$	Positive
`Mask pos4:`	CCG-C $\rightarrow$	Positive	`Mask pos1:`	TGG-C $\rightarrow$	Negative
`Mask pos5:`	CCGG- $\rightarrow$	Positive	`Mask pos1:`	TGGA- $\rightarrow$	Negative

Figure 5: Example sequences and corresponding mock predictions based on a predictive model is given. - denotes the masking operation where the bp is masked with a zero vector. (left) A sequence that does not change its prediction when a single bp is masked and (right) A sequence that has its prediction changed when a bp is masked. According to the discarding operation described in Section C, we retain the sequence on the left for experiments involving mutation while discarding the right one.

D Codon Frequency and the Impact of T Mutations

T mutations		A mutations		G mutations	
GAA	29.0	TAG	(already a stop codon)	TAA	(already a stop codon)
AAA	24.0	TAC	15.6	TTA	7.2
CAA	11.8	TAT	12.0	TCA	11.7
GGA	16.3	TGA	(already a stop codon)	TAC	15.6
AGA	11.5	TCA	11.7	TAA	(already a stop codon)
CGA	6.3	TTA	7.2	TAT	12.0
GAG	40.8	TGG	12.8		
AAG	32.9	TGC	12.2		
CAG	34.6	TGT	9.9		
		TCG	4.5		
		TTG	12.6		
Total	**207.2**	Total	**98.5**	Total	**46.5**

Figure 6: Frequency of various codons that can be mutated into a stop codon in the coding region are grouped for T, A, and G, original data taken from [3].

In the main paper we noted that the mutations in the first nt of each codon in the coding region were far more likely to cause a prediction change. Specifically, we observed that T mutations that occur in the first nt of codons were over-represented compared to A or G mutations (that occur in the second or the third nt). Upon a detailed inspection we notice that this topic is highly related to the frequency of codons that are readily mutable to stop codons in the coding region. In Figure 6, we provide the frequency of these codons (codon frequency in $1,000$ obtained from [3]) and observe that the number of codons that are readily mutable to one of the stop codons with T mutations is significantly higher than the other two, which explains this phenomenon.

E Mutations to the Beta-globin Gene

In Section 4.2 of the main paper, we conducted a case study on the HBB gene (also known as the Beta-globin gene) in order to examine the properties of our model with regards to mutation on a particular gene. The HBB gene directs the production of a protein called Beta-globin, which is a component of hemoglobin, which makes it one of the fundamental building blocks of blood. Hemoglobin consists of four protein substructures, two of them being Beta-globin and the other two being α-globin. Various mutations in the HBB gene may lead to a disease called Beta thalassemia which can be characterized as the measurable deficiency of Beta-globin chains in hemoglobin. As a result, this disease may lead to severe complications such as anemia.

E.1 Pathological Mutations

The comprehensive work of [11] characterized a variety of mutations that may lead to Beta thalassemia into three categories: mutations in the transcription process, mutations in RNA processing, and mutations in RNA translation. In order to evaluate the mutations detailed in [11], we take the functional HBB gene from Human Gene Bank and format it according to the convention in our paper (60 nt upstream + `ATG` + 140 nt downstream). Naturally, we cannot evaluate all those mutations due to limitations regarding the length of the sequence as well as positioning. The gene that we employ, after formatting, starts from the transcription initiation site and ends approximately around the middle of the second exon. Based on this sequence, we are able to perform 26 mutations detailed in [11].

Our model predicts that the functional HBB gene has a 95% likelihood to initiate translation. Based on this gene, we perform the aforementioned 26 mutations and display the results in Figure 7. This figure shows the reduction in the translation likelihood for the mutated sequences. Furthermore, we also display the origin as well as the precise position of the mutation. Note that, differently from the main text, we also conduct frame shift, insertion, and deletion mutations that are documented in [11]. Similar to the results obtained in the main text, we observe that when mutations occur relatively far away from the start codon, their impact lessens. Of all the mutations evaluated, CAP+40 to +43 (-`AAAC`) and CAP+10 (-T) have the most significant effect, followed by a variety of other mutations. These observations confirm that our model is able to identify that for the majority of the documented mutations the likelihood of translation initiation decreases, thus indicating the potential for gene-related issues. Despite this, not all known mutations that lead to Beta thalassemia lead to a TIS-negative prediction. This can be attributed to the fact that some of those mutations still lead to synthesized proteins (albeit dysfunctional ones), in other words, translation initiation is not an issue. However, making a clear distinction between correctness and incorrectness of predictions made due to mutations is not a simple task and a direction worthy of further exploration.

E.2 Benign single nucleotide polymorphisms

Above, we investigated a variety of mutations for the HBB gene that are discovered to carry pathological properties. While such mutations can cause disease or increase the risk of developing a certain condition, others are considered benign, meaning they do not have any significant impact on health. Among other things, this implies that they should not significantly affect translation initiation. Therefore, our goal in investigating these mutations is to observe whether our model's predictions will change significantly or not.

dbSNP, also known as the NCBI database of genetic variation [8], is a comprehensive public database of common and rare genetic variations found in humans and other organisms. This database is maintained by the National Center for Biotechnology Information (NCBI) and serves as a valuable resource for researchers studying genetic variation and its implications for human health and disease. The database also includes information on the frequency and distribution of these variations in different populations, as well as their association with various health conditions. Using dbSNP (build 155), we investigate known benign mutations of the HBB gene and find 33 of them that fall under the specifications of our data (i.e., mutations that are within 140bp of the translation initiation).

Using the final model as well as the bootstrapped models, we provide predictions obtained with sequences that contain these mutations in Figure 9 and Figure 10. As it can be seen, predictions obtained with our models indicate those mutations to be not detrimental to translation initiation and confirm the authenticity of our observations from another angle.

E.3 Investigating Remaining Mutations

Nevertheless, after performing the mutations documented in the [11], we continue with experimenting all possible point mutations similar to the experiment conducted in the main paper. Performing 600 point mutations on this gene, we identify 12 mutations that impact the likelihood of translation of which 4 are already documented in [11] (see Figure 8). Of those undocumented mutations, our model believes that the (HBB+25 $\texttt{A}\rightarrow\texttt{T}$) mutation will be the most significant one with this mutation having 86% likelihood of TIS-negative prediction.

E.4 Reproducibility of Observations

Reproducibility is one of the most important principles in scientific research, and it is especially crucial in bioinformatics research where computational tools are employed in conjunction with genomic, transcriptomic, proteomic, and metabolomic data. In this context, bootstrapping is particularly useful since it provides a way to estimate the variability of the results and the uncertainty of the statistical measures. Furthermore, bootstrapping can also help to detect potential sources of bias and errors in the analysis. By comparing the results obtained from the resampled data sets, we can identify whether our findings are consistent across different subsets of the data or whether they are sensitive to particular outliers or subsets.

Recall from Section B.2 that 50 additional models were trained with bootstrapping. Now, we employ these models to determine if the observations made with the final model can be replicated. Consequently, in Figure 7 and Figure 8, next to the predictions obtained from the final model, we provide the mean and the standard deviation of predictions obtained from 50 bootstrapped models. The data provided in Figure 7 are also visualized with boxplots in Figure 11. As it can be seen, for the majority of the observations, the final model and the bootstrapped average align. However, it is crucial to note that in certain cases, the results from the final model are slightly different, due to the bias inherent in the (unadjusted) bootstrap method, as described earlier. The bootstrap results support our main conclusion, however, and affirm that the results obtained by the final model hold up under replication.

Sequence	Final model	Bootstrap results
Original sequence:	95% positive	93 (±5)% positive
(Mediterranean) frame shift: CD1 (-G)	98% negative	99 (±1)% negative
(Mediterranean) frame shift: CD5 (-CT)	67% negative	79 (±19)% negative
(Mediterranean) frame shift: CD6 (-A)	86% negative	89 (±13)% negative
(Mediterranean) frame shift: CD8 (-AA)	74% negative	80 (±21)% negative
(Tunisian) frame shift: CD9 (+TA)	99% negative	97 (±4)% negative
(Malay) frame shift: CD15 (-T)	26% negative	71 (±25)% negative
(Asian Indian) frame shift: CD16 (-C)	41% negative	70 (±26)% negative
(Japanese) frame shift: CD26 (+T)	3.5% negative	5.1 (±6)% negative
(Egyptian) frame shift: CD28 (-C)	2.3% negative	5.2 (±6)% negative
(Chinese) frame shift: CD31 (-C)	4.9% negative	3 (±5)% negative
(Malay) frame shift: CD35 (-C)	4.9% negative	3 (±4)% negative
(Brazilian) non-sense: CD6 (G→ T)	96% negative	83 (±23)% negative
(English) non-sense: CD7 (G→ T)	80% negative	73 (±26)% negative
(Asian Indian, Japanese) non-sense: CD15 (G→ A)	83% negative	56 (±30)% negative
(Portuguese, Japanese) non-sense: CD15 (G→ A)	94% negative	54 (±28)% negative
(Chinese, Japanese) non-sense: CD17 (A→ T)	93% negative	56 (±27)% negative
(Reunion Island) non-sense: CD22 (G→ T)	15% negative	30 (±22)% negative
(Thai) non-sense: CD26 (G→ T)	13% negative	17 (±18)% negative
(Asian Indian) 5'UTR: CAP+1 (A→ C)	4.7% negative	3.5 (±4)% negative
(Chinese) 5'UTR: CAP+8 (C→ T)	4.9% negative	3.3 (±45% negative
(Bulgarian) 5'UTR: CAP+20 (C→ T)	4.8% negative	3.4 (±4)% negative
(Mediterranean, Bulgarian) 5'UTR: CAP+22 (G→ A)	8.5% negative	5.5 (±6)% negative
(Greek Cypriot) 5'UTR: CAP+33 (C→ G)	2.8% negative	2.9 (±4)% negative
(Italian) 5'UTR: CAP+45 (G→ C)	18% negative	8.4 (±7)% negative
(Greeks) 5'UTR: CAP+10 (-T)	100% negative	99 (±1)% negative
(Chinese) 5'UTR: CAP+40 to +43 (-AAAC)	100% negative	99 (±1)% negative

Figure 7: Predictions obtained from TISRover+ for (the first line) Beta-Globin gene and (rest) mutated variants of the same gene that cause Beta thalassemia disease. Mutations are accommodated by their type as well as their origin. In order to describe the mutations, we follow the notation of [11]. Bootstrap results are calculated using 50 models discussed in Section B.3

+19	G→ T	91% Reduction in translation likelihood (documented)
+22	G→ T	74% Reduction in translation likelihood (documented)
+47	G→ A	78% Reduction in translation likelihood (documented)
+48	G→ A	89% Reduction in translation likelihood (documented)
+52	A→ T	88% Reduction in translation likelihood (documented)
-21	T→ A	16% Reduction in translation likelihood (Avg. 2% reduction)
-3	A→ C	41% Reduction in translation likelihood (Avg. 18% reduction)
-3	A→ T	47% Reduction in translation likelihood (Avg. 20% reduction)
-2	C→ A	18% Reduction in translation likelihood (Avg. 3% reduction)
-2	C→ T	19% Reduction in translation likelihood (Avg. 4% reduction)
-1	C→ T	27% Reduction in translation likelihood (Avg. 5% reduction)
+25	A→ T	81% Reduction in translation likelihood (Avg. 65% reduction)

Figure 8: Mutations that are identified to be potentially problematic by TISRover+ for Beta-globin gene. Reduction in the translation likelihood is identified from our final model while the numbers in brackets are the averages calculated with 50 bootstrapped models discussed in Section B.3

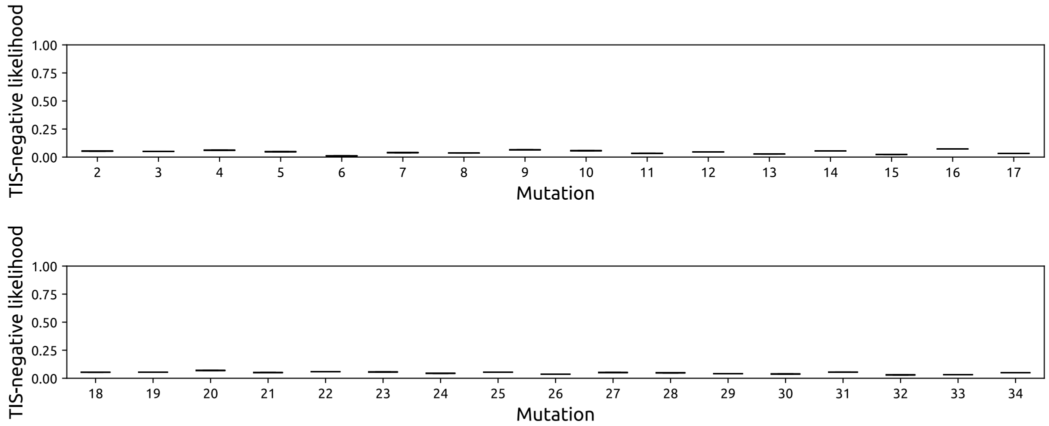

Figure 9: Predictions obtained from the final model for sequences that contain point mutations that are classified as "benign mutations" for the Beta-Globin gene.

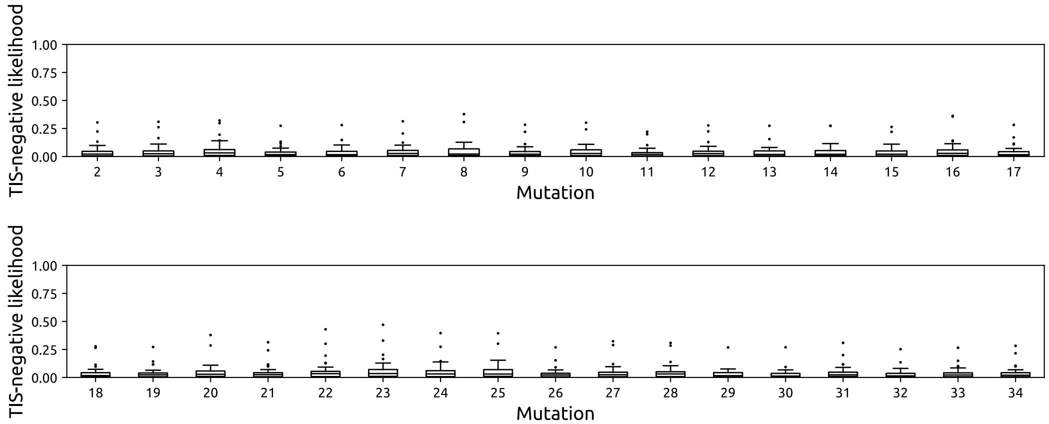

Figure 10: Predictions obtained from 50 bootstrapped models for sequences that contain point mutations that are classified as "benign mutations" for the Beta-Globin gene.

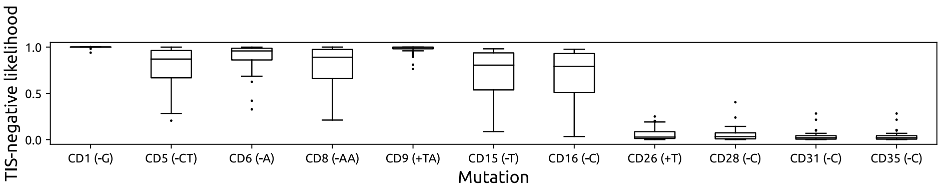

(a) Frameshift mutations

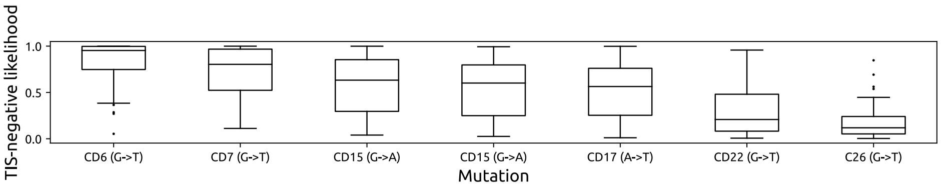

(b) Nonsense mutations

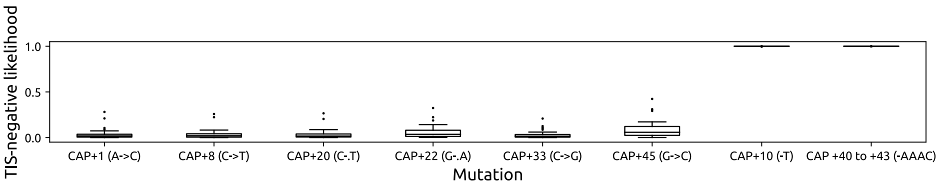

(c) Mutations in 5’UTR

Figure 11: Boxplots created using predictions of 50 bootstrapped models detailed in Section B.3 for mutation in the Beta-globin gene for (a) frameshift mutations, (b) nonsense mutations, and (c) mutations in 5’UTR. The results illustrated in the above figures correspond to the data provided in Figure 7.

F Mutations and the Reduction in the Likelihood Translation Initiation

In Section 4.1 of the main paper, we provided the impact of mutations on translation by measuring the change in the likelihood of translation initiation. In Figure 12, we provide a more detailed view of those changes. While Figure 12a shows the distribution of change in the likelihood, Figure 12b to Figure 12g provide a detailed overview for individual mutation categories. Note that different mutation categories have different distributions, indicating that not all mutations that cause a prediction change are the same in terms of reduction in the translation initiation likelihood. Overall, we observe that mutations grouped in Figure 12c and Figure 12e have the highest impact.

- **Mutations in the Kozak sequence** – We represent the mutations performed on the Kozak sequence that occur in the upstream (uKozak mutations) as well as downstream regions (dKozak mutations). As briefly shown in Figure 8 of the main paper, we observe that the mutations occurring in the downstream part of the Kozak sequence have a higher impact on the likelihood of translation initiation compared to the ones occurring in the upstream Kozak region (see Figure 12b and Figure 12c).
- **Upstream `ATG` mutations** – We observe that the mutations that lead to the creation of `ATG` triplets in the 5'UTR (`uATG`) have a greater effect on reducing translation initiation likelihood compared to other mutations that occur in 5'UTR. Also, this type of mutation accounts for the majority of the mutations in the upstream region.
- **Downstream stop codon mutations** – Mutations of this type create a stop codon (`TAA`, `TGA`, or `TAG`) in the downstream region which may lead to premature termination of translation. We observe that the mutations that create a stop codon in the downstream region cause the most severe effect on the translation initiation compared to other mutations. Also, since the majority of mutations are of this type, we can see that distributions in Figure 12e and the overall distribution displayed in Figure 12a are extremely similar.
- **Other mutations** – Besides the aforementioned mutations, we also provide the distributions of prediction change for other mutations that occur in the upstream and the downstream region in Figure 12f and Figure 12g, respectively. Note that these mutations are far less impactful on translation initiation compared to others.

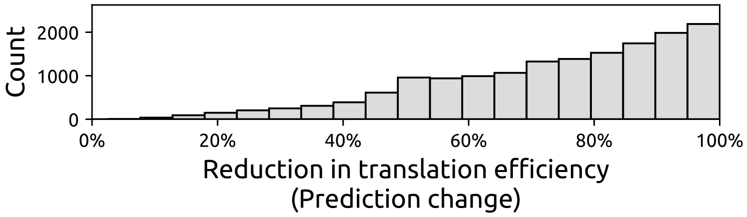

(a) All mutations

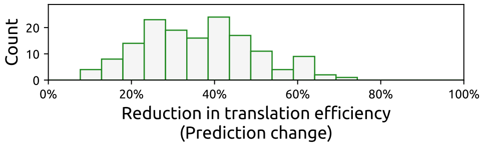

(b) uKozak mutations

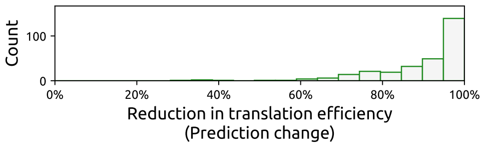

(c) dKozak mutations

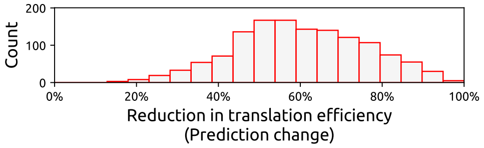

(d) uATG mutations

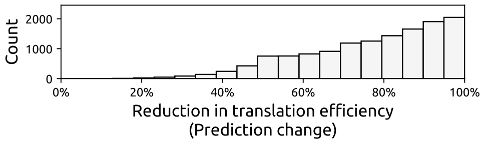

(e) dStop mutations

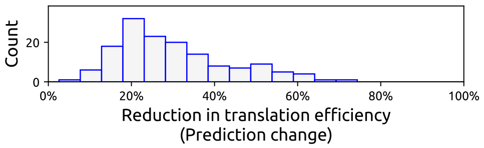

(f) uOther mutations

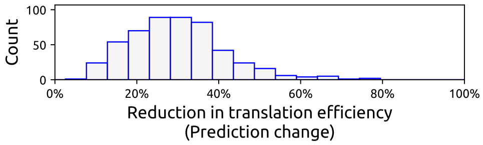

(g) dOther mutations

Figure 12: Distribution of reduction in translation efficiency for different mutation types: (a) all mutations, (b) mutations that occur at the Kozak sequence in the upstream region (sequence position -3, -2, -1 from the start codon), (c) mutations that occur at the Kozak sequence in the downstream region (sequence position +4 from the start codon), (d) mutations that led to the creation of an `ATG` triplet in the upstream region, (e) mutations that led to the creation of a stop codon (dStop, i.e., `TAA`, `TGA`, or `TAG`) in the downstream region, (f) mutations that occur in upstream region excluding `ATG` or Kozak sequence (-3, -2, -1 positions), (e) mutations that occur in downstream region excluding dStop or Kozak sequence (+4 position).

G Mutations in 5'UTR

In the main paper, we identified a select few non-ATG mutations in the 5'UTR that cause TIS-positive sequence to be predicted as a TIS-negative one. In Figure 13 and in Figure 14, we provide the frequency maps for bp that mutated and mutatations, respectively. In what follows, for those 149 non-ATG mutations in the 5'UTR, we list the original sequence, their mutated variant, and predictions obtained from our model. Note that a number of original (unmutated) sequences have multiple appearances, which means that there exists multiple mutations which lead to a prediction change for that particular sequence. In order to visualize the distribution of repetitions, we provide the histogram of sequence occurences in Figure 15. We believe that the existence of these sequences indicate that certain sequences are inherently unstable.

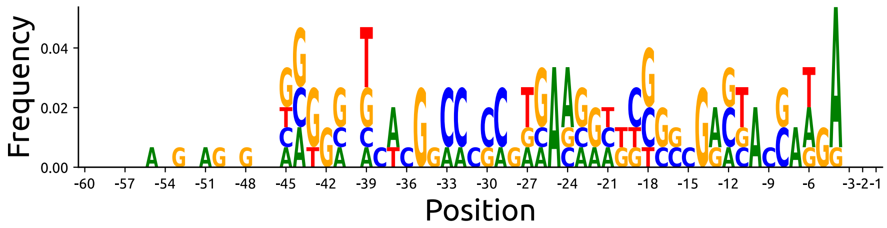

Figure 13: Frequency of bp in the 5'UTR that, when mutated, lead to a change in the prediction.

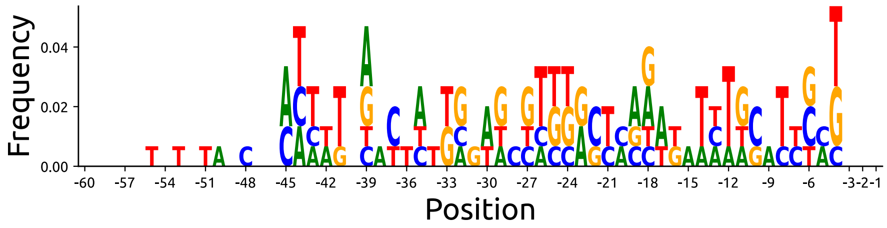

Figure 14: Frequency of mutations in the 5'UTR that cause a change in the prediction.

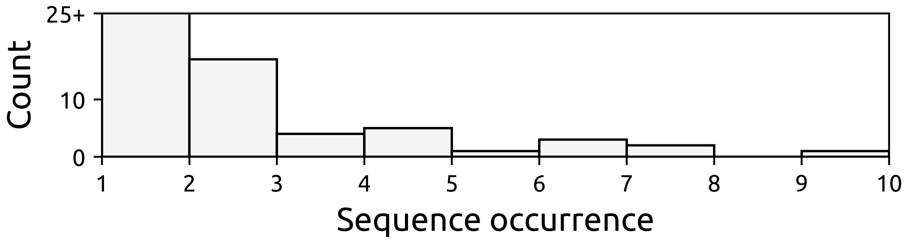

Figure 15: Histogram of occurrences for original (unmutated) sequences.

G.1 List of Impactful Mutations in 5'UTR

For each gene, the first sequence represents the original sequence, and the second sequence represents the mutated version with ATG denoting translation initiation.

EIF3H:
5′-CCTGATTCTAGGACAGGGATGGGGGTGCAGCACTGATCCAGGACCCAGAATGGAGGCATCATG → 98.7% TIS-positive
5′-CCTGATTCTAGGACAGGGATGGGGGTGCAGCACTGCTCCAGGACCCAGAATGGAGGCATCATG → 72.5% TIS-negative

EIF3H:
5′-CCTGATTCTAGGACAGGGATGGGGGTGCAGCACTGATCCAGGACCCAGAATGGAGGCATCATG → 98.7% TIS-positive
5′-CCTGATTCTAGGACAGGGATGGGGGTGCAGCACTGTTCCAGGACCCAGAATGGAGGCATCATG → 66.3% TIS-negative

EIF4G1:
5′-GGCCACCGTTTCCAGGGTTATATCCTGTGTTGTGACCTCATGGTTTAAGTGGGAATAAAGATG → 99.5% TIS-positive
5′-GGCCACCGTTTCCAGGGTTATATCCTGTGTTGTGACCTCATGGTTTACGTGGGAATAAAGATG → 65.2% TIS-negative

ZZZ3:
5′-CATTCTAGATCATAGCTCCAACTCCTTCAGGATATAAGGAAAAGAGATTATATTTCCACAATG → 85.3% TIS-positive
5′-CATTCTAGATCATAGCTCCAACTCCTTTAGGATATAAGGAAAAGAGATTATATTTCCACAATG → 76.3% TIS-negative

MAPK13:
5′-TCAGCCTCCCTAGTAGCTATGATTACAGGCGCATTGGAGTGACTGTCTGGCATCACCAAGATG → 92.8% TIS-positive
5′-TCAGCCTCCCTAGTAGCTATGATTACAGGCGCATTGGAGCGACTGTCTGGCATCACCAAGATG → 68.6% TIS-negative

IL25:
5′-GTAAAACTATTCATGCCTGGACAGTTTTTGACGGTGCAGTCTTGCTATATGGTGTGAGAAATG → 90.0% TIS-positive
5′-GTAAAACTATTCATGCCTGGACAGTTTTTGACGGTGCAGTCTTGCTATATGGTGTGTGAAATG → 70.3% TIS-negative

EIF3H:
5′-CCTGATTCTAGGACAGGGATGGGGGTGCAGCACTGATCCAGGACCCAGAATGGAGGCATCATG → 98.7% TIS-positive
5′-CCTGATTCTAGGACAGGGATGGGGGTGCAGCACCGATCCAGGACCCAGAATGGAGGCATCATG → 60.7% TIS-negative

EIF3H:
5′-CCTGATTCTAGGACAGGGATGGGGGTGCAGCACTGATCCAGGACCCAGAATGGAGGCATCATG → 98.7% TIS-positive
5′-CCTGATTCTAGGACAGGGATGGGGGTGCAGCACTCATCCAGGACCCAGAATGGAGGCATCATG → 59.2% TIS-negative

EIF3H:
5′-CCTGATTCTAGGACAGGGATGGGGGTGCAGCACTGATCCAGGACCCAGAATGGAGGCATCATG → 98.7% TIS-positive
5′-CCTGATTCTAGGACAGGGATGGGGGTGCAGCACTTATCCAGGACCCAGAATGGAGGCATCATG → 58.6% TIS-negative

IQCJ-SCHIP1:
5′-TCAGTAAAGACATTTGAAGAGCTGCGGCTAAAGGAAGAGTTACTAAAAGGAATCTATGCAATG → 91.1% TIS-positive
5′-TCAGTAAAGACATTTGAAGAGCTGCGGCTAAAGTAAGAGTTACTAAAAGGAATCTATGCAATG → 65.9% TIS-negative

LRRC15:
5′-TACTGCACCAGCTGAGCAATGCATGGAGTGGACCTGTAGGCGACTTGCATCGTCTTCAACATG → 99.5% TIS-positive
5′-TACTGCACCAGCTGAGCAATGCATGGAGTGGACCTGTACGCGACTTGCATCGTCTTCAACATG → 56.8% TIS-negative

KIFC3:
5′-TTTTTCTGCCCGCGGTGTCTCAGATTCATTCTTAAGGAACTGAGAACTTAATCTTCCAAAATG → 88.4% TIS-positive
5′-TTTTTCTGCCCGCGGTGTCTCAGATTCATTCTTAAGTAACTGAGAACTTAATCTTCCAAAATG → 66.4% TIS-negative

EIF3H:
5′-CCTGATTCTAGGACAGGGATGGGGGTGCAGCACTGATCCAGGACCCAGAATGGAGGCATCATG → 98.7% TIS-positive
5′-CCTGATTCTAGGACAGGGATGGGGGTGCAGCACTGGTCCAGGACCCAGAATGGAGGCATCATG → 55.7% TIS-negative

MATCAP2:
5′-CCCACCTCGGCCTCCCAAAGTACTGGGATTACAGGACTAATGGCTGAAGGATAAATCAACATG → 99.9% TIS-positive
5′-CCCACCTCGGCCTCCCAAAGTACTGGGATTACAGGACTAATGGCTGAAGGATACATCAACATG → 54.2% TIS-negative

INSIG1:
5′-CCCGGCGGCTACCCATGCCGAGGCACACGGAATGCAGTGCTGAACACGGAGGCGCGCACGATG → 95.8% TIS-positive
5′-CCCGGCGGCTACCCATGCCGAGGCACACGGAATGCAGTGCTCAACACGGAGGCGCGCACGATG → 56.6% TIS-negative

ZZZ3:
5′-CATTCTAGATCATAGCTCCAACTCCTTCAGGATATAAGGAAAAGAGATTATATTTCCACAATG → 85.3% TIS-positive
5′-CATTCTAGATCATAGCTCCAACTCCTTCAGGATATAAGGTAAAGAGATTATATTTCCACAATG → 67.0% TIS-negative

EIF4G1:
5′-GGCCACCGTTTCCAGGGTTATATCCTGTGTTGTGACCTCATGGTTTAAGTGGGAATAAAGATG → 99.5% TIS-positive
5′-GGCCACCGTTTCCAGGGTTATATCCTGTGTTGTGACCTCATGGTTTATGTGGGAATAAAGATG → 52.6% TIS-negative

IL25:
5′-GTAAAACTATTCATGCCTGGACAGTTTTTGACGGTGCAGTCTTGCTATATGGTGTGAGAAATG → 90.0% TIS-positive
5′-GTAAAACTATTCATGCCTGGACAGTTTTTGACGGTGCAGTCTTGCTATATGGTGCGAGAAATG → 61.8% TIS-negative

IL25:
5′-GTAAAACTATTCATGCCTGGACAGTTTTTGACGGTGCAGTCTTGCTATATGGTGTGAGAAATG → 90.0% TIS-positive
5′-GTAAAACTATTCATGCCTGGACAGTTTTTGACGGTGCAGTCTTGCTATATGGTGTGGGAAATG → 61.5% TIS-negative

MATCAP2:
5′-CCCACCTCGGCCTCCCAAAGTACTGGGATTACAGGACTAATGGCTGAAGGATAAATCAACATG $\rightarrow$ 99.9% TIS-positive
5′-CCCACCTCGGCCTCCCAAAGTACTGGGATTACAGGACTAATGGCTGAAGGATATATCAACATG $\rightarrow$ 50.4% TIS-negative

SMIM14:
5′-AGAAATTTAGCCATAAAATTCCATCCAAAACTTCCACTGAATTGGAGTGAAAGGGAAGCAATG $\rightarrow$ 73.5% TIS-positive
5′-AGAAATTTAGCCATAAAATTCCATCCAAAACTTCCACTGAATTGGAGTGAAAGGTAAGCAATG $\rightarrow$ 76.0% TIS-negative

MIF4GD:
5′-GGGGACGGCGGCGGCGCGGCCCGCGTGACCCAGCGCCGCGCCCCGTGGCCCCGCCGCAGGATG $\rightarrow$ 91.6% TIS-positive
5′-GGGGACGGCGACGGCGCGGCCCGCGTGACCCAGCGCCGCGCCCCGTGGCCCCGCCGCAGGATG $\rightarrow$ 57.1% TIS-negative

IL25:
5′-GTAAAACTATTCATGCCTGGACAGTTTTTGACGGTGCAGTCTTGCTATATGGTGTGAGAAATG $\rightarrow$ 90.0% TIS-positive
5′-GTAAAACTATTCATGCCTGGACAGTTTTTGACGGTGCAGTCTTGCTATATGGTGTCAGAAATG $\rightarrow$ 57.4% TIS-negative

RCCD1:
5′-GCTGGACGAATAAATGGCTAGATAAGCCAGGCGGCGGCCGGCAGAGGGCGCTGCTCGGGCATG $\rightarrow$ 80.1% TIS-positive
5′-GCTGGACGAATAAATGGCTAGGTAAGCCAGGCGGCGGCCGGCAGAGGGCGCTGCTCGGGCATG $\rightarrow$ 66.8% TIS-negative

PHLDB2:
5′-TTGTCAAACTCAACAAATTGAAGGTTAACACCTTAAGAGTTGTAGTTACTGACCAGAAATATG $\rightarrow$ 70.7% TIS-positive
5′-TTGTCAAACTCAACAAATTGAAGGTTAACACCTTAAGAGTAGTAGTTACTGACCAGAAATATG $\rightarrow$ 76.0% TIS-negative

ATXN7L3:
5′-CGTGTGTCTGGCGTGTGGAGCTAGTGCTCCCGCTTATTCGCCACTGTCGCCACCGCTGTCATG $\rightarrow$ 81.2% TIS-positive
5′-CGTGTGTCTGGCGTGTGGAGCAAGTGCTCCCGCTTATTCGCCACTGTCGCCACCGCTGTCATG $\rightarrow$ 64.6% TIS-negative

IL25:
5′-GTAAAACTATTCATGCCTGGACAGTTTTTGACGGTGCAGTCTTGCTATATGGTGTGAGAAATG $\rightarrow$ 90.0% TIS-positive
5′-GTAAAACTATTCATGCCTGGACAGTTTTTGACGGTGCAGTCTTGCTATATGGTGGGAGAAATG $\rightarrow$ 55.8% TIS-negative

IL25:
5′-GTAAAACTATTCATGCCTGGACAGTTTTTGACGGTGCAGTCTTGCTATATGGTGTGAGAAATG $\rightarrow$ 90.0% TIS-positive
5′-GTAAAACTATTCATGCCTGGACAGTTTTTGACGGTGCAGTCTTGCTATATGGTGTGCGAAATG $\rightarrow$ 54.8% TIS-negative

MIF4GD:
5′-GGGGACGGCGGCGGCGCGGCCCGCGTGACCCAGCGCCGCGCCCCGTGGCCCCGCCGCAGGATG $\rightarrow$ 91.6% TIS-positive
5′-GGGGACGGCGGCGGCGCGGCCCGCGTGACCAAGCGCCGCGCCCCGTGGCCCCGCCGCAGGATG $\rightarrow$ 53.1% TIS-negative

EYA1:
5′-TTGAGGCCATAGTGGGAGGCCCAGTGGATCCTGAGAAGCTAGCCCGGGCAGTGAGTGTGGATG $\rightarrow$ 83.3% TIS-positive
5′-TTGAGGCCATAGTGGGAGGCCCAGTGGATCCTGGGAAGCTAGCCCGGGCAGTGAGTGTGGATG $\rightarrow$ 60.2% TIS-negative

SCN4B:
5′-GAGAAACTGGATAGAAAAGCTCCAAGTCGTATAAACAACCCAGAACTTTTGGGACAATATATG $\rightarrow$ 70.4% TIS-positive
5′-GAGAAACTGGATAGAAAAGCTCCAAGTCGTATAAACAACCCATAACTTTTGGGACAATATATG $\rightarrow$ 72.7% TIS-negative

MIF4GD:
5′-GGGGACGGCGGCGGCGCGGCCCGCGTGACCCAGCGCCGCGCCCCGTGGCCCCGCCGCAGGATG $\rightarrow$ 91.6% TIS-positive
5′-GGGGACGGCGGCGGCGTGGCCCGCGTGACCCAGCGCCGCGCCCCGTGGCCCCGCCGCAGGATG $\rightarrow$ 51.1% TIS-negative

CEP131:
5′-GCGCGCCAGGCAGGCTTCGCGGCTGCGCCCCCGGCCCGCGCAGGACCTGCCTTGTCCACCATG $\rightarrow$ 85.4% TIS-positive
5′-GCGCGCCAGGCAGGCTTCGCGGCTGCGCCCCTGGCCCGCGCAGGACCTGCCTTGTCCACCATG $\rightarrow$ 56.5% TIS-negative

CHMP6:
5′-ACCGACATCGTGCTGCAGCTGGACGACATCCAGCATGCCATCGCCATCAACTATGACCCAATG $\rightarrow$ 89.9% TIS-positive
5′-ACCGACATCGTGCTGCAGCTGGACGACGTCCAGCATGCCATCGCCATCAACTATGACCCAATG $\rightarrow$ 51.0% TIS-negative

TGM5:
5′-CTTCTCACAGGAGATTGCTTCCATTTGATCTGAAAGCTATACATACAAGGGGCTAGAAGCATG $\rightarrow$ 79.4% TIS-positive
5′-CTTCTCACAGGAGATTGCTTCCATTTGATCTGAAAGCTATAGATACAAGGGGCTAGAAGCATG $\rightarrow$ 61.1% TIS-negative

ACBD5:
5′-AGGTGGACAAGCCCTATGGGCTAAGACAGAGGGTCCTCAGAAAGGAGTGCGGACGCCGTCATG $\rightarrow$ 81.3% TIS-positive
5′-AGGTGGACAAGCCCTATGGGCTACGACAGAGGGTCCTCAGAAAGGAGTGCGGACGCCGTCATG $\rightarrow$ 57.5% TIS-negative

C1QTNF7:
5′-TGCGCACACTAGAGTTCAAAGACTCCAGCTGTTGGAAGGTCTTGTGAGCAATAGCCAAAGATG → 82.5% TIS-positive
5′-TGCGCACACTAGAGTTCAAAGACTCCAGGTGTTGGAAGGTCTTGTGAGCAATAGCCAAAGATG → 55.6% TIS-negative

ACBD5:
5′-AGGTGGACAAGCCCTATGGGCTAAGACAGAGGGTCCTCAGAAAGGAGTGCGGACGCCGTCATG → 81.3% TIS-positive
5′-AGGTGGACAAGCCCTATGGGCTATGACAGAGGGTCCTCAGAAAGGAGTGCGGACGCCGTCATG → 56.6% TIS-negative

RNF8:
5′-CAGCCAGAACCTAGGTCAGGGTCTCGCTCGGTGCTGACCGCCCCCGGGGTCGAGTAGGCGATG → 82.7% TIS-positive
5′-CAGCCAGAACCTAGGTCAGGGTCTCGCTCGGTGCTGACCGCCCCCGTGGTCGAGTAGGCGATG → 54.7% TIS-negative

RNF8:
5′-CAGCCAGAACCTAGGTCAGGGTCTCGCTCGGTGCTGACCGCCCCCGGGGTCGAGTAGGCGATG → 82.7% TIS-positive
5′-CAGCCAGAACCTAGGTCAGGGTCTCGCTCGGTGCTGACCGCCCCCGGGGTCGAGTATGCGATG → 54.5% TIS-negative

A1CF:
5′-CACCAGGAAGAAGCCGTCAGCGGCGTGGGCAGCGGCAGATGGGCCACGCGAGGAGCCCACATG → 85.7% TIS-positive
5′-CACCAGGAAGAAGCCATCAGCGGCGTGGGCAGCGGCAGATGGGCCACGCGAGGAGCCCACATG → 51.3% TIS-negative

C1QTNF7:
5′-GGGCGTCCCGGGATATTTGGAGGATAAAGGTGTGGGCAAGAATGTCTTTTCCGCAGGGTGATG → 68.7% TIS-positive
5′-GGGCGTCCCGGGATATTTGGAGGATAAAGGTGTGGGTAAGAATGTCTTTTCCGCAGGGTGATG → 68.3% TIS-negative

SCML4:
5′-AGTGACGTCCTTGTCAGCCATGCCGAGTGCAGAGCGCCTACTGTATTGTAGGCATTTGAAATG → 86.7% TIS-positive
5′-AGTGACGTCCTTGTCAGCCATGCCGAGTGCAGAGTGCCTACTGTATTGTAGGCATTTGAAATG → 50.1% TIS-negative

PPFIA2:
5′-CCGCGTTCCTATGGACAGACGCACAGACACCTGCAGGAGTGACACTGCTGATCCTGTAACATG → 82.6% TIS-positive
5′-CCGCGTTCCTATGGACAGACGCACAGACACCTGCAGGAGTGACACTGCTGGTCCTGTAACATG → 53.6% TIS-negative

PAM16:
5′-CTCCCCTCTCGGCGGCTGGCAGTCCTTGCCTCTGCCCCGCCTTCCAGATGCTTTGGAGTCATG → 83.4% TIS-positive
5′-CTCCCCTCTCGGCGGCTGGCAGTCCTTGCCTCTGCCCCGCCTTCCATATGCTTTGGAGTCATG → 52.7% TIS-negative

TEPSIN:
5′-GTCGTTTCCATCCCCTTTGGGGGGCTGCGGATTCACCCCTGTGGGAAATGGCACCTCATCATG → 80.4% TIS-positive
5′-GTCGTTTCCATCCCCTTTGGGGGGCTGGGGATTCACCCCTGTGGGAAATGGCACCTCATCATG → 55.6% TIS-negative

GZMB:
5′-GCTTCTGGCTCCAGACCGCCCTCCGGATCGGACCCTGCGAATGGTTTTGGCTATATCTTCATG → 73.0% TIS-positive
5′-GCTTCTGGCTCCAGACCGCCCTCCGGATCGGACCCTGCGAATGGTTTTTGCTATATCTTCATG → 62.5% TIS-negative

CADPS:
5′-TAACCCACCAGTGACACCACTCTTCCCGGCTGGATGGTCAATTAGCTCTGGCATGAGAGAATG → 82.0% TIS-positive
5′-TAACCCACCAGTGACACCACTCTTCCCGGCTGGATGGTCAATGAGCTCTGGCATGAGAGAATG → 53.4% TIS-negative

MCOLN2:
5′-ACTTTGAAGTTGCCATATCCTGACTGAAGTCCTTCCCAGAACGGCAGTAGTTGGTCGAGTATG → 83.3% TIS-positive
5′-ACTTTGAAGTTGCCATATCCTGACTCAAGTCCTTCCCAGAACGGCAGTAGTTGGTCGAGTATG → 51.7% TIS-negative

ACBD5:
5′-AGGTGGACAAGCCCTATGGGCTAAGACAGAGGGTCCTCAGAAAGGAGTGCGGACGCCGTCATG → 81.3% TIS-positive
5′-AGGTGGACAAGCCCTATGGGCGAAGACAGAGGGTCCTCAGAAAGGAGTGCGGACGCCGTCATG → 53.6% TIS-negative

TMEM116:
5′-AGGAGAAGTGGAGTTTGGAAGTTCAGGGGCACAGGGGCACAGGCCCACGACTGCAGCGGGATG → 80.9% TIS-positive
5′-AGGAGAAGTGGAGTTTGGAAGTTCAGGGGCACAGGGGCACAGGCCCACGACTGCAACGGGATG → 53.2% TIS-negative

ACBD5:
5′-AGGTGGACAAGCCCTATGGGCTAAGACAGAGGGTCCTCAGAAAGGAGTGCGGACGCCGTCATG → 81.3% TIS-positive
5′-AGGTGGACAAGCCCTATGGGCAAAGACAGAGGGTCCTCAGAAAGGAGTGCGGACGCCGTCATG → 52.5% TIS-negative

OSBPL1A:
5′-TGAGCATGGAGAGCTTAGCTTGGACAGCCAGCTGATGAGGGACACATCGAATCAAGATACATG → 80.5% TIS-positive
5′-TGAGCATGGAGAGCTAAGCTTGGACAGCCAGCTGATGAGGGACACATCGAATCAAGATACATG → 53.2% TIS-negative

OSBPL1A:
5′-TGAGCATGGAGAGCTTAGCTTGGACAGCCAGCTGATGAGGGACACATCGAATCAAGATACATG → 80.5% TIS-positive
5′-TGAGCATGGAGAGCTTTGCTTGGACAGCCAGCTGATGAGGGACACATCGAATCAAGATACATG → 53.1% TIS-negative

OSBPL1A:
5′-TGAGCATGGAGAGCTTAGCTTGGACAGCCAGCTGATGAGGGACACATCGAATCAAGATACATG → 80.5% TIS-positive
5′-TGAGCATGGAGAGCTTCGCTTGGACAGCCAGCTGATGAGGGACACATCGAATCAAGATACATG → 52.5% TIS-negative

CYB5R3:
5′-ACAGGAAAGTGGAACAAGGTCCTTGTGCTCCTTTTTCCAGGTGCTTCCAGAGGCAGGGCTATG → 77.0% TIS-positive
5′-ACAGGTAAGTGGAACAAGGTCCTTGTGCTCCTTTTTCCAGGTGCTTCCAGAGGCAGGGCTATG → 55.9% TIS-negative

CFAP65:
5′-AGCCCGGGCAGAAGGGCGGTTGCTGCAGCTTTGGCCGAGGTTCGGGCTCGAACTTGGAAAATG → 68.9% TIS-positive
5′-AGCCCGGGCAGAAGGGCGGTTGCTGCAGCTTTGGCCGAGGTACGGGCTCGAACTTGGAAAATG → 63.8% TIS-negative

RAB14:
5′-TTGGGCCGGGCGCGGTGGCTTACTCCTGTAATCCCAGCACTTTGGGAAGCTGAGTTTGTGATG → 72.3% TIS-positive
5′-TTGGGCCGGGCGCGGTGGCTTACTCCTGTAATCCCAGCACTTTGGGAAGGTGAGTTTGTGATG → 60.2% TIS-negative

SAMRCD2:
5′-GAGTGGACTCGACGCGGAGCCCGGAGTCCGGATCGCGGCACCGCGGGACGGGACGGAGCGATG → 76.6% TIS-positive
5′-GAGTGGACTCGACGCGGAGCCCGGAGTCCGGATCGCGGCACCGCGGGATGGGACGGAGCGATG → 55.5% TIS-negative

OSBPL1A:
5′-TGAGCATGGAGAGCTTAGCTTGGACAGCCAGCTGATGAGGGACACATCGAATCAAGATACATG → 80.5% TIS-positive
5′-TGAGCATGGAGAGCTTATCTTGGACAGCCAGCTGATGAGGGACACATCGAATCAAGATACATG → 51.5% TIS-negative

C1orf185:
5′-TGCTAAGCTCCAGGTCCTGAGATTAAATTAGGGGCTGGAGCTCACTGCACTCCAGCAGTCATG → 79.9% TIS-positive
5′-TGCTAAGCTCCAGGTACTGAGATTAAATTAGGGGCTGGAGCTCACTGCACTCCAGCAGTCATG → 51.6% TIS-negative

QARS1:
5′-CAAGCATGGAAATTGTGAATTGTGTGTGTGGCCAGACCAGTACCTCCAAGTGTTCAGAAGATG → 76.6% TIS-positive
5′-CAAGCATGGAAATTGTGAATTGTGTGTGTGGGCAGACCAGTACCTCCAAGTGTTCAGAAGATG → 54.5% TIS-negative

PCED1B:
5′-TGACCATCCGCAGAGCCATCCTGTGCAAAGGAAGGAGCTAGGCTGTGCGCCCTGGGCGTCATG → 74.6% TIS-positive
5′-TGACCATCCGCAGAGCCATCCTGTGCAAAGGAAGGAGGTAGGCTGTGCGCCCTGGGCGTCATG → 56.4% TIS-negative

RPS6KL1:
5′-GGAGTAAGCAATGATATGCTGTTTAGATATGCTGAAAAGATAACGAGGAAATATTCTGAAATG → 79.6% TIS-positive
5′-GGAGTAAGCAATGATATGCTGTTTAGATATGCTGAAAAGATAACGAGGTAATATTCTGAAATG → 51.3% TIS-negative

EIF2S1:
5′-GCGATACCTAGGGCGGAAGTGCTCTCGGCGGAAGTGATCGCTGTGTGAATCGTGGGTGGGATG → 76.6% TIS-positive
5′-GCGATACCTAGGGCGGAAGTGCTCTCGGCGTAAGTGATCGCTGTGTGAATCGTGGGTGGGATG → 54.0% TIS-negative

PCED1B:
5′-TGACCATCCGCAGAGCCATCCTGTGCAAAGGAAGGAGCTAGGCTGTGCGCCCTGGGCGTCATG → 74.6% TIS-positive
5′-TGACCATCCGCAGAGCCATCCTGTGCAAAGGAAGGAGCTACGCTGTGCGCCCTGGGCGTCATG → 55.7% TIS-negative

QARS1:
5′-CAAGCATGGAAATTGTGAATTGTGTGTGTGGCCAGACCAGTACCTCCAAGTGTTCAGAAGATG → 76.6% TIS-positive
5′-CAAGCATGGTAATTGTGAATTGTGTGTGTGGCCAGACCAGTACCTCCAAGTGTTCAGAAGATG → 53.6% TIS-negative

TGM5:
5′-CTTCTCACAGGAGATTGCTTCCATTTGATCTGAAAGCTATACATACAAGGGGCTAGAAGCATG → 79.4% TIS-positive
5′-CTTCTCACAGGAGATTGCTTCCATTTGATCTGAAAGCTATAAATACAAGGGGCTAGAAGCATG → 50.7% TIS-negative

KNSTRN:
5′-CGGCGGGGTTTCTTCGTTGCATTGCCTGAGAGGAGCGGAGTCTGCCAGGTGGTGTCCATCATG → 78.3% TIS-positive
5′-CGGCGGGGTTTCTTCGTTGCATTGCCTGAGAGGAGCGGGGTCTGCCAGGTGGTGTCCATCATG → 51.6% TIS-negative

GZMB:
5′-GCTTCTGGCTCCAGACCGCCCTCCGGATCGGACCCTGCGAATGGTTTTGGCTATATCTTCATG → 73.0% TIS-positive
5′-GCTTCTGGCTCCAGACCGCCCTCCGGATAGGACCCTGCGAATGGTTTTGGCTATATCTTCATG → 56.9% TIS-negative

SLC39A12:
5′-GGAGCTCTGCCCCTAGATCTTTGCACAGCTGACTTCAGGCTGGACCGCCAGCTGGAGACGATG → 67.3% TIS-positive
5′-GGAGCTCTGCCCCTAGATCTTTGCACAGCTGACTTCAGGCTGGACCGCAAGCTGGAGACGATG → 62.7% TIS-negative

SCN4B:
5′-GAGAAACTGGATAGAAAAGCTCCAAGTCGTATAAACAACCCAGAACTTTTGGGACAATATATG → 70.4% TIS-positive
5′-GAGAAACTGGATAGAAAAGCTCCAAGTCGTATAAACAACCCAGAACTTTAGGGACAATATATG → 59.5% TIS-negative

HK2:
5′-TCCGGCCCGGGGCGGGCGAAGAGGGAGCGAGTGGGCGGGGCCGCGTGGCGTCAGCGCAAGATG → 66.9% TIS-positive
5′-TCCGGCCCGGGGCGGGCGAAGAGGGAGTGAGTGGGCGGGGCCGCGTGGCGTCAGCGCAAGATG → 62.5% TIS-negative

RIC3:
5′-CTCATTGTTCTGTCATCTGAGGTGGGCATCTCTGAGCTGAGGCTGGAGAGACTACTCCAAATG → 77.5% TIS-positive
5′-CTCATTGTTCTGTCATCTGAGGTGGGCATCTCTGAGCTGAGGCTGGAGAGCCTACTCCAAATG → 51.6% TIS-negative

KNSTRN:
5′-CGGCGGGGTTTCTTCGTTGCATTGCCTGAGAGGAGCGGAGTCTGCCAGGTGGTGTCCATCATG → 78.3% TIS-positive
5′-CGGCGGGGTTTCTTCGTTGCATTGCCTGAGAGGAGCGGAGTCTACCAGGTGGTGTCCATCATG → 50.4% TIS-negative

ROBO3:
5′-AGGCAGGGAAATGATACAGATAAGACTCAGGGTGCACAGACTTGTGTGGCCAACGGTGTAATG → 75.9% TIS-positive
5′-AGGCAGGGAAATGATACAGGTAAGACTCAGGGTGCACAGACTTGTGTGGCCAACGGTGTAATG → 52.4% TIS-negative

EIF2S1:
5′-GCGATACCTAGGGCGGAAGTGCTCTCGGCGGAAGTGATCGCTGTGTGAATCGTGGGTGGGATG → 76.6% TIS-positive
5′-GCGATACCTAGGGCGGAAGTGCTCTCGGCGGAAGTGATCGCTGTGTTAATCGTGGGTGGGATG → 51.4% TIS-negative

LIMA1:
5′-ACAGGGATACGGTACTGTCCATGGACCTCTGAGCTGCCCTTTCCACATCGCTGATTCACAATG → 75.2% TIS-positive
5′-ACAGGGATACGGTACTGTCCATGGACCTCTGAGCTGCCCTTTCCACATCGCTCATTCACAATG → 52.5% TIS-negative

TNFAIP3:
5′-GCCTGCACGCCCCCTGCCGAGTACAGTCCCGCGCAGCGCCAGTGCCTGAGCCCGGAAGAGATG → 62.5% TIS-positive
5′-GCCTGCACGCCCCCTGCCGAGTACAGTCCCGCGCAGCGCCAGTGCCTGAGCCTGGAAGAGATG → 65.2% TIS-negative

LIMA1:
5′-ACAGGGATACGGTACTGTCCATGGACCTCTGAGCTGCCCTTTCCACATCGCTGATTCACAATG → 75.2% TIS-positive
5′-ACAGGGATACGGTACTGTCCATGGACCTCTGAGCTGCCCTTTCCACATCGCTTATTCACAATG → 52.3% TIS-negative

SMIM14:
5′-AGAAATTTAGCCATAAAATTCCATCCAAAACTTCCACTGAATTGGAGTGAAAGGGAAGCAATG → 73.5% TIS-positive
5′-AGAAATTTAGCCATAAAATTCCATCCAAAACTTCCACTGAATTGGAGTTAAAGGGAAGCAATG → 53.9% TIS-negative

KCNIP2:
5′-TGTGTGCTTACTCCCTGACCAGATGCTGCGGTGCACGGGGCAGCCACCATCTCTGCATGCATG → 74.2% TIS-positive
5′-TGTGTGCTTACTCCCTCACCAGATGCTGCGGTGCACGGGGCAGCCACCATCTCTGCATGCATG → 52.6% TIS-negative

KCNIP2:
5′-TGTGTGCTTACTCCCTGACCAGATGCTGCGGTGCACGGGGCAGCCACCATCTCTGCATGCATG → 74.2% TIS-positive
5′-TGTGTGCTTACTCCCTTACCAGATGCTGCGGTGCACGGGGCAGCCACCATCTCTGCATGCATG → 52.5% TIS-negative

CFAP65:
5′-AGCCCGGGCAGAAGGGCGGTTGCTGCAGCTTTGGCCGAGGTTCGGGCTCGAACTTGGAAAATG → 68.9% TIS-positive
5′-AGCCCGGGCAGAAGGGCGGTTGCTGCAGCTTTGGCCGAGGTTGGGGCTCGAACTTGGAAAATG → 57.6% TIS-negative

SEC22C:
5′-AAGAACAGAAATACAGGTGGGTCACCTAGGCTGCAGCCAGACTCCAGAGCTTTGCACTACATG → 74.5% TIS-positive
5′-AAGAACAGAAATACAGGTGGGTAACCTAGGCTGCAGCCAGACTCCAGAGCTTTGCACTACATG → 51.7% TIS-negative

PTGR2:
5′-TACCGGTGGCGGCGCGGAGGCAGAAGGCGGTGTCCGAGTAGGGGCCTCTGCCCCACCAGGATG → 75.5% TIS-positive
5′-TACCGGTGGCGGCGCGGAGGCAGAAGGCGGTGTCCGGGTAGGGGCCTCTGCCCCACCAGGATG → 50.7% TIS-negative

FUZ:
5′-TGGACTGGTGATGACAGGCTCTTCAGCTCCCTGCAAGTGACCGGGCCTGGGGAACAGGGCATG → 71.6% TIS-positive
5′-TGGACTGGTGATGACCGGCTCTTCAGCTCCCTGCAAGTGACCGGGCCTGGGGAACAGGGCATG → 54.5% TIS-negative

NUP107:
5′-GGGCGGTCCCGGGAATCGCACAGGACCCGGCTCCCCTCGCCTAAGCTGCTGGGGGGCGCCATG → 75.2% TIS-positive
5′-GGGCGGTCCCGGGAATCGCACAGGACCCGGCTCCCCTCGTCTAAGCTGCTGGGGGGCGCCATG → 50.2% TIS-negative

PHLDB2:
5′-TTGTCAAACTCAACAAATTGAAGGTTAACACCTTAAGAGTTGTAGTTACTGACCAGAAATATG → 70.7% TIS-positive
5′-TTGTCAAACTCAACAAATTTAAGGTTAACACCTTAAGAGTTGTAGTTACTGACCAGAAATATG → 54.4% TIS-negative

PDILT:
5′-CTGAAGGAAAGAGAACTTGATCAGCAGAAGTTGTGGCTACAGCAAAGCATCAAAAATGTGATG → 69.5% TIS-positive
5′-CTGAAGGAAAGAGAACTTGATCAGTAGAAGTTGTGGCTACAGCAAAGCATCAAAAATGTGATG → 55.6% TIS-negative

SCN4B:
5′-GAGAAACTGGATAGAAAAGCTCCAAGTCGTATAAACAACCCAGAACTTTTGGGACAATATATG → 70.4% TIS-positive
5′-GAGAAACTGGATAGAAAAGCTCCAAGTCGTATAAACAACCCACAACTTTTGGGACAATATATG → 54.7% TIS-negative

PHLDB2:
5′-TTGTCAAACTCAACAAATTGAAGGTTAACACCTTAAGAGTTGTAGTTACTGACCAGAAATATG → 70.7% TIS-positive
5′-TTGTCAAACTCAACAAATTGAAGGTTAACACCTTAAGGGTTGTAGTTACTGACCAGAAATATG → 54.2% TIS-negative

PCED1B:
5′-TGACCATCCGCAGAGCCATCCTGTGCAAAGGAAGGAGCTAGGCTGTGCGCCCTGGGCGTCATG → 74.6% TIS-positive
5′-TGACCATCCGCAGAGCCATCCTGTGCAAAGGAAGGTGCTAGGCTGTGCGCCCTGGGCGTCATG → 50.2% TIS-negative

SCN4B:
5′-GAGAAACTGGATAGAAAAGCTCCAAGTCGTATAAACAACCCAGAACTTTTGGGACAATATATG → 70.4% TIS-positive
5′-GAGAAACTGGATAGAAAAGCTCCAAGTCGTATAAACAACCCAAAACTTTTGGGACAATATATG → 54.5% TIS-negative

FUZ:
5′-TGGACTGGTGATGACAGGCTCTTCAGCTCCCTGCAAGTGACCGGGCCTGGGGAACAGGGCATG → 71.6% TIS-positive
5′-TGGACTGGTGATGACAAGCTCTTCAGCTCCCTGCAAGTGACCGGGCCTGGGGAACAGGGCATG → 53.1% TIS-negative

PCED1B:
5′-TGACCATCCGCAGAGCCATCCTGTGCAAAGGAAGGAGCTAGGCTGTGCGCCCTGGGCGTCATG → 74.6% TIS-positive
5′-TGACCATCCGCAGAGCCATCCTGTGCAAAGGAAGGGGCTAGGCTGTGCGCCCTGGGCGTCATG → 50.1% TIS-negative

PCED1B:
5′-TGACCATCCGCAGAGCCATCCTGTGCAAAGGAAGGAGCTAGGCTGTGCGCCCTGGGCGTCATG → 74.6% TIS-positive
5′-TGACCATCCGCACAGCCATCCTGTGCAAAGGAAGGAGCTAGGCTGTGCGCCCTGGGCGTCATG → 50.1% TIS-negative

PHLDB2:
5′-TTGTCAAACTCAACAAATTGAAGGTTAACACCTTAAGAGTTGTAGTTACTGACCAGAAATATG → 70.7% TIS-positive
5′-TTGTCAAACTCAACAAATTGAAGGTTAACACCTTAAGAGTTGTAGTTACGGACCAGAAATATG → 53.2% TIS-negative

STK25:
5′-AGCCCCTACATCACCCGCTACTTTGGCTCCTACCTAAAGAGCACCAAGCTATGGATCATCATG → 73.5% TIS-positive
5′-AGCCCCTACATCACCCGCTACTTTGGCTCCTACCTAAAGAGCACCAAGCTATGGCTCATCATG → 50.4% TIS-negative

KLRC1:
5′-GTCTTGCCTGCGCCCCTTTGGCACCCCCTGCCCACCTCTAAGCCCCAGCGACGAGCAGAGATG → 71.8% TIS-positive
5′-GTCTTGCCTGCGCCCCTTTGGCACCCCCTGCCCACCTCTAAGCCCAAGCGACGAGCAGAGATG → 52.0% TIS-negative

SLC39A12:
5′-GGAGCTCTGCCCCTAGATCTTTGCACAGCTGACTTCAGGCTGGACCGCCAGCTGGAGACGATG → 67.3% TIS-positive
5′-GGAGCTCTGCCCCTAGATCTTTGCACAGCTGACTTCAAGCTGGACCGCCAGCTGGAGACGATG → 56.4% TIS-negative

ZNF385B:
5′-AGGTGCTGGCAAATCCATTATCTGATGAGGGTCCTGAGGGAGTCTAACAAGTTAGCAGAAATG → 64.9% TIS-positive
5′-AGGTGCTGGCAAATCCATTATCTGATGAGGGTCCTGCGGGAGTCTAACAAGTTAGCAGAAATG → 58.8% TIS-negative

CFAP65:
5′-AGCCCGGGCAGAAGGGCGGTTGCTGCAGCTTTGGCCGAGGTTCGGGCTCGAACTTGGAAAATG → 68.9% TIS-positive
5′-AGCCCGGGCAGAAGGGCGGTTGCTGCAGCTTTGGCCGAGGTTCGGGCTCGCACTTGGAAAATG → 54.6% TIS-negative

CFAP65:
5′-AGCCCGGGCAGAAGGGCGGTTGCTGCAGCTTTGGCCGAGGTTCGGGCTCGAACTTGGAAAATG → 68.9% TIS-positive
5′-AGCCCGGGCAGAAGGGCGGTTGCTGCAGCTTTGGCCGAGGTTCGTGCTCGAACTTGGAAAATG → 54.2% TIS-negative

CFAP65:
5′-AGCCCGGGCAGAAGGGCGGTTGCTGCAGCTTTGGCCGAGGTTCGGGCTCGAACTTGGAAAATG → 68.9% TIS-positive
5′-AGCCCGGGCAGAAGGGCGGTTGCTGCAGCTTTGGCCGAGGTTAGGGCTCGAACTTGGAAAATG → 54.1% TIS-negative

PDILT:
5′-CTGAAGGAAAGAGAACTTGATCAGCAGAAGTTGTGGCTACAGCAAAGCATCAAAAATGTGATG → 69.5% TIS-positive
5′-CTGAAGGAAAGAGAACTTTATCAGCAGAAGTTGTGGCTACAGCAAAGCATCAAAAATGTGATG → 53.3% TIS-negative

NAP1L4:
5′-CTGCTTCCACGAGGCCTGTCAGTCAGTGCCCCACTTGTGACTGAGTGTGCAGTGCCCAGCATG → 71.9% TIS-positive
5′-CTGCTTCCACGAGGCCTGTCAGTCAGTGCCCCACTTGTGACTGAGTATGCAGTGCCCAGCATG → 50.7% TIS-negative

IRF5:
5′-CCCTGATGGCCTCTTCCTGCAGATGCCCTGCTCTCTGACCTGGAGACTACCACCTCGCACATG → 65.9% TIS-positive
5′-CCCTGATGGCCTCTTCCTGCACATGCCCTGCTCTCTGACCTGGAGACTACCACCTCGCACATG → 56.7% TIS-negative

KLRC1:
5′-GTCTTGCCTGCGCCCCTTTGGCACCCCCTGCCCACCTCTAAGCCCCAGCGACGAGCAGAGATG → 71.8% TIS-positive
5′-GTCTTGCCTGCGCCCCTTTGGCACCCCCTGCCCACCTCTAAGCCGCAGCGACGAGCAGAGATG → 50.7% TIS-negative

SRSF4:
5′-CTGTCCTGACGGTGCACACACTGCTGTGTGGAGGAACAGAGGAGTCCAGCTGGCCTTCACATG → 72.3% TIS-positive
5′-CTGTCCTGACGGTGCACACACTGCTGTGTGGGGGAACAGAGGAGTCCAGCTGGCCTTCACATG → 50.2% TIS-negative

PDILT:
5′-CTGAAGGAAAGAGAACTTGATCAGCAGAAGTTGTGGCTACAGCAAAGCATCAAAAATGTGATG → 69.5% TIS-positive
5′-CTGAAGGAAAGAGAACTTGATAAGCAGAAGTTGTGGCTACAGCAAAGCATCAAAAATGTGATG → 52.7% TIS-negative

CACNA2D2:
5′-GCTGCTGCTGCAGTGGGAGGAGCCCTGGCAGTGGGGGCTGTGCCCGTGGTGCTCAGTGCCATG → 67.9% TIS-positive
5′-GCTGCTGCTGCAGTGGGAGTAGCCCTGGCAGTGGGGGCTGTGCCCGTGGTGCTCAGTGCCATG → 54.0% TIS-negative

MYH2:
5′-AAAGCTCTGTCTTCTGCAGGCATCCTGTCGGCTCACAAGATAATAGCCAGCTCCCCAGAAATG → 70.5% TIS-positive
5′-AAAGCTCTGTCTTCTGCAGGCATCCTGTCGGCTCACAAGATAATAGCCATCTCCCCAGAAATG → 51.4% TIS-negative

PDILT:
5′-CTGAAGGAAAGAGAACTTGATCAGCAGAAGTTGTGGCTACAGCAAAGCATCAAAAATGTGATG → 69.5% TIS-positive
5′-CTGAAGGAAAGAGAACTTGATCAGCAGAAGTTGGGGCTACAGCAAAGCATCAAAAATGTGATG → 52.2% TIS-negative

RDH5:
5′-CTAGAAGAAGTGGAAACCACTAAAGAAGCTGGGGAAACTGTGCCCCCCGACACCTCCAAGATG → 66.9% TIS-positive
5′-CTAGAAGAAGTGGAAACCACTAAAGAAGGTGGGGAAACTGTGCCCCCCGACACCTCCAAGATG → 54.8% TIS-negative

FUZ:
5′-TGGACTGGTGATGACAGGCTCTTCAGCTCCCTGCAAGTGACCGGGCCTGGGGAACAGGGCATG → 71.6% TIS-positive
5′-TGGACTGGTGATGACAGGCTCTTCAACTCCCTGCAAGTGACCGGGCCTGGGGAACAGGGCATG → 50.1% TIS-negative

ZNF385B:
5′-AGGTGCTGGCAAATCCATTATCTGATGAGGGTCCTGAGGGAGTCTAACAAGTTAGCAGAAATG → 64.9% TIS-positive
5′-AGGTGCTGGCAAATCCATTATCTGATGAGGGTCCTGAGGGAGTCTAACAAGTTAGCGGAAATG → 56.8% TIS-negative

CFAP65:
5′-AGCCCGGGCAGAAGGGCGGTTGCTGCAGCTTTGGCCGAGGTTCGGGCTCGAACTTGGAAAATG → 68.9% TIS-positive
5′-AGCCCGGGCAGAAGGGCAGTTGCTGCAGCTTTGGCCGAGGTTCGGGCTCGAACTTGGAAAATG → 52.6% TIS-negative

PDILT:
5′-CTGAAGGAAAGAGAACTTGATCAGCAGAAGTTGTGGCTACAGCAAAGCATCAAAAATGTGATG → 69.5% TIS-positive
5′-CTGAAGGAAAGAGAACTTGATCAGCAGACGTTGTGGCTACAGCAAAGCATCAAAAATGTGATG → 51.8% TIS-negative

CFAP65:
5′-AGCCCGGGCAGAAGGGCGGTTGCTGCAGCTTTGGCCGAGGTTCGGGCTCGAACTTGGAAAATG → 68.9% TIS-positive
5′-AGCCCGGGCAGAAGGGCTGTTGCTGCAGCTTTGGCCGAGGTTCGGGCTCGAACTTGGAAAATG → 51.9% TIS-negative

ZNF385B:
5′-AGGTGCTGGCAAATCCATTATCTGATGAGGGTCCTGAGGGAGTCTAACAAGTTAGCAGAAATG → 64.9% TIS-positive
5′-AGGTGCTGGCAAATCCATTATCTGATGAGGGTCCTGAGGGAGTCTAACAAGTTAGCTGAAATG → 55.5% TIS-negative

TNFAIP3:
5′-GCCTGCACGCCCCCTGCCGAGTACAGTCCCGCGCAGCGCCAGTGCCTGAGCCCGGAAGAGATG → 62.5% TIS-positive
5′-GCCTGCACGCCCCCTGCCGAGTACAGTCCCGAGCAGCGCCAGTGCCTGAGCCCGGAAGAGATG → 57.8% TIS-negative

PDILT:
5′-CTGAAGGAAAGAGAACTTGATCAGCAGAAGTTGTGGCTACAGCAAAGCATCAAAAATGTGATG → 69.5% TIS-positive
5′-CTGAAGGAAAGAGAACTCGATCAGCAGAAGTTGTGGCTACAGCAAAGCATCAAAAATGTGATG → 50.6% TIS-negative

SPRED3:
5′-TCCAGGTGCTGTGTGCGGGCCCTCTCCATACAGAGGCTGTGGTACTTCTGGTTCCTTCTGATG → 69.6% TIS-positive
5′-TCCAGGTGCTGTGTGCGGGCCCTCTCCATACAGAAGCTGTGGTACTTCTGGTTCCTTCTGATG → 50.3% TIS-negative

PDILT:
5′-CTGAAGGAAAGAGAACTTGATCAGCAGAAGTTGTGGCTACAGCAAAGCATCAAAAATGTGATG → 69.5% TIS-positive
5′-CTGAAGGAAAGAGAACTTGATCAGCATAAGTTGTGGCTACAGCAAAGCATCAAAAATGTGATG → 50.3% TIS-negative

ZNF385B:
5′-AGGTGCTGGCAAATCCATTATCTGATGAGGGTCCTGAGGGAGTCTAACAAGTTAGCAGAAATG → 64.9% TIS-positive
5′-AGGTGCTGGCAAATCCATTATCTGATGAGGGTCCTGGGGGAGTCTAACAAGTTAGCAGAAATG → 54.9% TIS-negative

CACNA2D2:
5′-GCTGCTGCTGCAGTGGGAGGAGCCCTGGCAGTGGGGGCTGTGCCCGTGGTGCTCAGTGCCATG → 67.9% TIS-positive
5′-GCTGCTGCTGCAGTGGGAGGAGCCCTGGCAGTGGGGGCTGTGCCCGTGGTGCTCGGTGCCATG → 51.7% TIS-negative

TNFAIP3:
5′-GCCTGCACGCCCCCTGCCGAGTACAGTCCCGCGCAGCGCCAGTGCCTGAGCCCGGAAGAGATG → 62.5% TIS-positive
5′-GCCTGCACGCCCCCTGCCGAGTACAGTCCCGCGCAGCGCCAGTGCCTGAGCACGGAAGAGATG → 57.1% TIS-negative

CEP104:
5′-GACGAGTACGACGGAGAGGGGAATGAGGAGCAGAAGGGGCCCCCGGAGGGCTCAGAGACCATG → 64.5% TIS-positive
5′-GACGAGTACGACGGAGAGGGGAATGAGGAGCAGAAGGGGCCCCCGGAGGGCTTAGAGACCATG → 54.7% TIS-negative

IRF5:
5′-CCCTGATGGCCTCTTCCTGCAGATGCCCTGCTCTCTGACCTGGAGACTACCACCTCGCACATG → 65.9% TIS-positive
5′-CCCTGATGGCCTCTTCCTGCATATGCCCTGCTCTCTGACCTGGAGACTACCACCTCGCACATG → 53.3% TIS-negative

ZNF385B:
5′-AGGTGCTGGCAAATCCATTATCTGATGAGGGTCCTGAGGGAGTCTAACAAGTTAGCAGAAATG → 64.9% TIS-positive
5′-AGGTGCTGGCAAATCCATTATCTGATGAGGGTCCTGAAGGAGTCTAACAAGTTAGCAGAAATG → 54.0% TIS-negative

ZNF385B:
5′-AGGTGCTGGCAAATCCATTATCTGATGAGGGTCCTGAGGGAGTCTAACAAGTTAGCAGAAATG → 64.9% TIS-positive
5′-AGGTGCTGGCAAATCCATTATCTGATGAGGGTCCTGAGGGAGTATAACAAGTTAGCAGAAATG → 53.9% TIS-negative

SMTN:
5′-AGTAAAAGCCAGATAAAGCTCCATACTCAGAGTGCTCCCTTTGGACTGTGTCCCAAGGACATG → 60.0% TIS-positive
5′-AGTAAAAGCCAGATAAAGCTCCATACTCAGAGTGCTCCCTTTGGACTATGTCCCAAGGACATG → 58.7% TIS-negative

TNFAIP3:
5′-GCCTGCACGCCCCCTGCCGAGTACAGTCCCGCGCAGCGCCAGTGCCTGAGCCCGGAAGAGATG → 62.5% TIS-positive
5′-GCCTGCACGCCCCCTGACGAGTACAGTCCCGCGCAGCGCCAGTGCCTGAGCCCGGAAGAGATG → 56.2% TIS-negative

SLC39A12:
5′-GGAGCTCTGCCCCTAGATCTTTGCACAGCTGACTTCAGGCTGGACCGCCAGCTGGAGACGATG → 67.3% TIS-positive
5′-GGAGCTCTGCCCCTACATCTTTGCACAGCTGACTTCAGGCTGGACCGCCAGCTGGAGACGATG → 51.3% TIS-negative

SLC39A12:
5′-GGAGCTCTGCCCCTAGATCTTTGCACAGCTGACTTCAGGCTGGACCGCCAGCTGGAGACGATG → 67.3% TIS-positive
5′-GGAGCTCTGCCCCTAGATCTTTGCACAGCTGACTTCAGCCTGGACCGCCAGCTGGAGACGATG → 51.1% TIS-negative

IRF5:
5′-CCCTGATGGCCTCTTCCTGCAGATGCCCTGCTCTCTGACCTGGAGACTACCACCTCGCACATG → 65.9% TIS-positive
5′-CCCTGATGGCCTCTTCCTGTAGATGCCCTGCTCTCTGACCTGGAGACTACCACCTCGCACATG → 52.5% TIS-negative

FRMD3:
5′-GTAGAGCGGAGCCTGGCGGGCGTGGGAACCCAGGCCCCGCCGAGGCGGCCAGGAGGTGAGATG → 61.9% TIS-positive
5′-GTAGAGCGGAGCCTGGCGGGCGTGGTAACCCAGGCCCCGCCGAGGCGGCCAGGAGGTGAGATG → 56.0% TIS-negative

TNFAIP3:
5′-GCCTGCACGCCCCCTGCCGAGTACAGTCCCGCGCAGCGCCAGTGCCTGAGCCCGGAAGAGATG → 62.5% TIS-positive
5′-GCCTGCACGCCCCCTGCCGAGTACAATCCCGCGCAGCGCCAGTGCCTGAGCCCGGAAGAGATG → 55.0% TIS-negative

TNFAIP3:
5′-GCCTGCACGCCCCCTGCCGAGTACAGTCCCGCGCAGCGCCAGTGCCTGAGCCCGGAAGAGATG → 62.5% TIS-positive
5′-GCCTGCACGCCCCCTGCCGAGTACAGTCCGGCGCAGCGCCAGTGCCTGAGCCCGGAAGAGATG → 54.8% TIS-negative

CEP104:
5′-GACGAGTACGACGGAGAGGGGAATGAGGAGCAGAAGGGGCCCCCGGAGGGCTCAGAGACCATG → 64.5% TIS-positive
5′-GACGAGTACGACGGAGAGGGGAACGAGGAGCAGAAGGGGCCCCCGGAGGGCTCAGAGACCATG → 52.3% TIS-negative

FRMD3:
5′-GTAGAGCGGAGCCTGGCGGGCGTGGGAACCCAGGCCCCGCCGAGGCGGCCAGGAGGTGAGATG → 61.9% TIS-positive
5′-GTAGAGCGGAGCCTGGCGGGCGTGGGAACCAAGGCCCCGCCGAGGCGGCCAGGAGGTGAGATG → 53.2% TIS-negative

TNFAIP3:
5′-GCCTGCACGCCCCCTGCCGAGTACAGTCCCGCGCAGCGCCAGTGCCTGAGCCCGGAAGAGATG → 62.5% TIS-positive
5′-GCCTGCACGCCCCCTGCCGAGTACAGTCCCGCCCAGCGCCAGTGCCTGAGCCCGGAAGAGATG → 51.1% TIS-negative

TNFAIP3:
5′-GCCTGCACGCCCCCTGCCGAGTACAGTCCCGCGCAGCGCCAGTGCCTGAGCCCGGAAGAGATG → 62.5% TIS-positive
5′-GCCTGCACGCCCCCTGCCAAGTACAGTCCCGCGCAGCGCCAGTGCCTGAGCCCGGAAGAGATG → 51.1% TIS-negative

TNFAIP3:
5′-GCCTGCACGCCCCCTGCCGAGTACAGTCCCGCGCAGCGCCAGTGCCTGAGCCCGGAAGAGATG → 62.5% TIS-positive
5′-GCCTGCACGCCCCCTGCCGAGTACAGTCCCGCGCTGCGCCAGTGCCTGAGCCCGGAAGAGATG → 50.9% TIS-negative

FRMD3:
5′-GTAGAGCGGAGCCTGGCGGGCGTGGGAACCCAGGCCCCGCCGAGGCGGCCAGGAGGTGAGATG → 61.9% TIS-positive
5′-GTAGAGCTGAGCCTGGCGGGCGTGGGAACCCAGGCCCCGCCGAGGCGGCCAGGAGGTGAGATG → 51.3% TIS-negative

SMTN:
5′-AGTAAAAGCCAGATAAAGCTCCATACTCAGAGTGCTCCCTTTGGACTGTGTCCCAAGGACATG → 60.0% TIS-positive
5′-AGTAAAAGCCAGATAAAGCTCCATACTCAGAGTGCTCCCTTTGTACTGTGTCCCAAGGACATG → 53.0% TIS-negative

E2F5:
5′-AGCCCGCTCGGCCGCAGCTGCGCGCCTGTAGCCGCAGGTGGCTGCGATGGGACGGAAGCCATG → 58.9% TIS-positive
5′-AGCCCGCTCGGCCGCAGCTGCGCGCCTGTAGCCGCAGGTGGCTGCGATGGGACGGAGGCCATG → 51.8% TIS-negative

E2F5:
5′-AGCCCGCTCGGCCGCAGCTGCGCGCCTGTAGCCGCAGGTGGCTGCGATGGGACGGAAGCCATG → 58.9% TIS-positive
5′-AGCCCGCTCGGCCGCAGCTGCGCGCCTGTAGCCGCAGGTGGCTGCGATGGGACGGATGCCATG → 50.6% TIS-negative

References

[1] Bradley Efron and Robert Tibshirani. *An Introduction to the Bootstrap*. Number 57 in Monographs on Statistics and Applied Probability. Chapman & Hall, New York, 1993.

[2] Adam Frankish, Mark Diekhans, Irwin Jungreis, Julien Lagarde, Jane E Loveland, Jonathan M Mudge, Cristina Sisu, James C Wright, Joel Armstrong, If Barnes, et al. Gencode 2021. *Nucleic acids research*, 49(D1):D916–D923, 2021.

[3] GenScript. Codon frequency table. *https://www.ncbi.nlm.nih.gov/assembly/GCA_000001405.28*, 2022.

[4] Chuan Guo, Geoff Pleiss, Yu Sun, and Kilian Q Weinberger. On calibration of modern neural networks. In *International conference on machine learning*, pages 1321–1330. PMLR, 2017.

[5] Kaiming He, Xiangyu Zhang, Shaoqing Ren, and Jian Sun. Deep residual learning for image recognition. In *Proceedings of the IEEE Conference on Computer Vision and Pattern Recognition*, 2016.

[6] Yvan Saeys, Thomas Abeel, Sven Degroeve, and Yves Van de Peer. Translation initiation site prediction on a genomic scale: beauty in simplicity. *Bioinformatics*, 23(13):i418–i423, 2007.

[7] Valerie A Schneider, Tina Graves-Lindsay, Kerstin Howe, Nathan Bouk, Hsiu-Chuan Chen, Paul A Kitts, Terence D Murphy, Kim D Pruitt, Françoise Thibaud-Nissen, Derek Albracht,

et al. Evaluation of GRCh38 and de novo haploid genome assemblies demonstrates the enduring quality of the reference assembly. *Genome Research*, 27(5):849–864, 2017.

[8] Stephen T Sherry, M-H Ward, M Kholodov, J Baker, Lon Phan, Elizabeth M Smigielski, and Karl Sirotkin. dbsnp: the ncbi database of genetic variation. *Nucleic acids research*, 29(1):308–311, 2001.

[9] Karen Simonyan and Andrew Zisserman. Very deep convolutional networks for large-scale image recognition. *International Conference on Learning Representations*, 2015.

[10] Nitish Srivastava, Geoffrey Hinton, Alex Krizhevsky, Ilya Sutskever, and Ruslan Salakhutdinov. Dropout: A simple way to prevent neural networks from overfitting. *The Journal of Machine Learning Research*, 2014.

[11] Swee Lay Thein. The molecular basis of β-thalassemia. *Cold Spring Harbor perspectives in medicine*, 3(5):a011700, 2013.
